# Pulmonary infection by SARS-CoV-2 induces senescence accompanied by an inflammatory phenotype in severe COVID-19: possible implications for viral mutagenesis

**DOI:** 10.1183/13993003.02951-2021

**Published:** 2022-08-18

**Authors:** Konstantinos Evangelou, Dimitris Veroutis, Koralia Paschalaki, Periklis G. Foukas, Nefeli Lagopati, Marios Dimitriou, Angelos Papaspyropoulos, Bindu Konda, Orsalia Hazapis, Aikaterini Polyzou, Sophia Havaki, Athanassios Kotsinas, Christos Kittas, Athanasios G. Tzioufas, Laurence de Leval, Demetris Vassilakos, Sotirios Tsiodras, Barry R. Stripp, Argyris Papantonis, Giovanni Blandino, Ioannis Karakasiliotis, Peter J. Barnes, Vassilis G. Gorgoulis

**Affiliations:** 1 Molecular Carcinogenesis Group, Dept of Histology and Embryology, Medical School, National and Kapodistrian University of Athens, Athens, Greece; 2 Biomedical Research Foundation, Academy of Athens, Athens, Greece; 3 National Heart and Lung Institute, Imperial College London, London, UK; 4 2nd Dept of Pathology, Attikon University Hospital, Medical School, National and Kapodistrian University of Athens, Athens, Greece; 5 Laboratory of Biology, Dept of Medicine, Democritus University of Thrace, Alexandroupolis, Greece; 6 Lung and Regenerative Medicine Institutes, Cedars-Sinai Medical Center, Los Angeles, CA, USA; 7 Faculty of Health and Medical Sciences, University of Surrey, Guildford, UK; 8 Dept of Pathophysiology, Medical School, National and Kapodistrian University of Athens, Athens, Greece; 9 Institute of Pathology, Lausanne University Hospital, Lausanne, Switzerland; 10 4th Dept of Internal Medicine, Attikon University Hospital, University of Athens Medical School, Athens, Greece; 11 Hellenic Centre for Disease Control and Prevention, Athens, Greece; 12 Translational Epigenetics Group, Institute of Pathology, University Medical Center Göttingen, Göttingen, Germany; 13 Center for Molecular Medicine, University of Cologne, Cologne, Germany; 14 Oncogenomic and Epigenetic Unit, IRCCS, Regina Elena National Cancer Institute, Rome, Italy; 15 Faculty Institute for Cancer Sciences, Manchester Academic Health Sciences Centre, University of Manchester, Manchester, UK; 16 Center for New Biotechnologies and Precision Medicine, Medical School, National and Kapodistrian University of Athens, Athens, Greece; 17 Contributed equally; 18 P.J. Barnes and V.G. Gorgoulis contributed equally to this article as lead authors and supervised the work

## Abstract

**Background:**

Severe acute respiratory syndrome coronavirus 2 (SARS-CoV-2) infection of the respiratory system can progress to a multisystemic disease with aberrant inflammatory response. Cellular senescence promotes chronic inflammation, named senescence-associated secretory phenotype (SASP). We investigated whether coronavirus disease 2019 (COVID-19) is associated with cellular senescence and SASP.

**Methods:**

Autopsy lung tissue samples from 11 COVID-19 patients and 43 age-matched non-COVID-19 controls with similar comorbidities were analysed by immunohistochemistry for SARS-CoV-2, markers of senescence and key SASP cytokines. Virally induced senescence was functionally recapitulated *in vitro*, by infecting epithelial Vero-E6 cells and a three-dimensional alveosphere system of alveolar type 2 (AT2) cells with SARS-CoV-2 strains isolated from COVID-19 patients.

**Results:**

SARS-CoV-2 was detected by immunocytochemistry and electron microscopy predominantly in AT2 cells. Infected AT2 cells expressed angiotensin-converting enzyme 2 and exhibited increased senescence (p16^INK4A^ and SenTraGor positivity) and interleukin (IL)-1β and IL-6 expression. *In vitro*, infection of Vero-E6 cells with SARS-CoV-2 induced senescence (SenTraGor), DNA damage (γ-H2AX) and increased cytokine (IL-1β, IL-6, CXCL8) and apolipoprotein B mRNA-editing (APOBEC) enzyme expression. Next-generation sequencing analysis of progenies obtained from infected/senescent Vero-E6 cells demonstrated APOBEC-mediated SARS-CoV-2 mutations. Dissemination of the SARS-CoV-2-infection and senescence was confirmed in extrapulmonary sites (kidney and liver) of a COVID-19 patient.

**Conclusions:**

We demonstrate that in severe COVID-19, AT2 cells infected by SARS-CoV-2 exhibit senescence and a proinflammatory phenotype. *In vitro*, SARS-CoV-2 infection induces senescence and inflammation. Importantly, infected senescent cells may act as a source of SARS-CoV-2 mutagenesis mediated by APOBEC enzymes. Therefore, SARS-CoV-2-induced senescence may be an important molecular mechanism of severe COVID-19, disease persistence and mutagenesis.

## Abstract 2

**In severe COVID-19, alveolar type 2 (AT2) cells infected by SARS-CoV-2 exhibit senescence accompanied by a proinflammatory phenotype, a molecular mechanism that may be important in persistence of disease (post-acute sequelae of COVID-19) and mutagenesis** https://bit.ly/3fnopg9

## Introduction

The severe acute respiratory syndrome coronavirus 2 (SARS-CoV-2) causes coronavirus disease 2019 (COVID-19), which primarily affects the respiratory system. The clinical course ranges from asymptomatic to life-threatening respiratory failure accompanied by multisystemic inflammatory disease [1, 2]. Systemic disease may occur through a virally mediated inflammatory response that consists of a variety of proinflammatory cytokines and chemokines, including interleukin (IL)-1β, IL-6, IL-12, interferon-γ, tumour necrosis factor (TNF)-α, CXCL-8, CXCL-10 and CCL-2 [3, 4]. The link between viral infection of cells and development of severe lung disease and systemic manifestations is still poorly understood. Viral infection results in the activation of complex innate and adaptive immune responses that are orchestrated sequentially, involving several cell types and inflammatory mediators [5, 6]. At the cellular level, intrinsic defence mechanisms are activated and outcomes range from complete recovery to cell death [7–11]. An “intermediate” and essential cellular state that is overlooked, due to lack of efficient methodological tools, is cellular senescence [12, 13].

Cellular senescence is a stress response mechanism that preserves homeostasis. Senescent cells are characterised by prolonged and generally irreversible cell-cycle arrest and resistance to apoptosis [12, 14]. Additionally, they exhibit secretory features, collectively described as the senescence-associated secretory phenotype (SASP) [12]. SASP includes a variety of cytokines, chemokines, growth factors, proteases and other molecules, depending on the type of senescence and the cells involved [12, 15]. These inflammatory proteins are released in the extracellular space as soluble factors, transmembrane proteins following ectodomain shedding or as molecules engulfed within extracellular vesicles [16–18]. Cellular senescence plays a key role in several lung diseases, involving the senescence of several cell types in the lung [19, 20]. Under physiological conditions, senescence is transiently activated and SASP mediates the recruitment of immune cells for senescent cell clearance. In addition, other SASP factors promote tissue regeneration and repair, overall ensuring cellular/tissue homeostasis. In contrast, persistence of senescent cells exerts harmful properties promoting tissue dysfunction and the maintenance of chronic inflammation, *via* paracrine and systemic SASP [12, 15].

There is little published evidence linking viral infection to cellular senescence [21–26]. Given the implication of an inflammatory response in the progression of COVID-19 and SASP secretion by senescent cells, we investigated whether cellular senescence occurs in COVID-19. Since individual markers are not adequate to unequivocally detect senescence, especially *in vivo*, as they may also be present in nonsenescent conditions, we followed in a clinical setting a detailed multimarker algorithmic approach that we and others published recently and which was approved by the senescence community [12, 27]. We were the first to provide evidence supporting the proof for senescence in COVID-19-infected lung tissue by applying this algorithm in a prior, preprint version of the current manuscript [28], which has been confirmed by others subsequently [29]. This is the largest clinical study verifying viral-induced senescence by SARS-CoV-2 infection linked to a proinflammatory phenotype, which may contribute to acute and chronic clinical manifestations in severe COVID-19. Importantly, we also provide novel evidence for the generation of viral mutations promoted by SARS-CoV-2 persistence in senescent cells.

## Materials and methods

### Tissues

Formalin-fixed and paraffin-embedded autopsy lung tissue samples were obtained from 11 patients who died from severe COVID-19 (confirmed by reverse transcription (RT) quantitative (q)PCR) (table 1 and supplementary table S1a). Lung parenchyma displaying analogous lesions (atelectasis, fibrosis and infiltration by immune cells) from 43 age-matched patients with similar comorbidities from a previously published cohort [30] and new cases, resected prior to the COVID-19 outbreak, were used for comparison with COVID-19 samples for all experiments detailed herein (negative controls) (table 1 and supplementary table S1b). A non-COVID cohort of 60 age-matched lung samples was used as biological negative controls for validation of the anti-SARS-CoV-2 (G2) monoclonal antibody (supplementary material). Moreover, renal and liver tissue from one of the 11 COVID-19 cases and five non-COVID-19 patients were also available and were analysed for the presence of the virus and cellular senescence. Clinical sample collection and experimental use were approved by the Commission Cantonale D'éthique de la Recherche (University of Lausanne, Switzerland; 2020–01257) and the Bio-Ethics Committee of the University of Athens Medical School, Greece.

**TABLE 1:** Summary of clinical characteristics of patients

	**COVID-19**	**Controls^#^**	**Non-COVID-19 pneumonia^#,¶^**
**Participants**	11	25	18
**Male/female**	8/3	17/8	10/8
**Age (years)**	71±5	70±2	75±1
**Smokers (current or ex-smokers)**	3	25	18
**Acute pneumonia**	11	0	18
**Comorbidities**			
COPD	4	14	0
Cardiovascular disease^+^	7	9	18
Diabetes	4	2	8
Chronic kidney disease	2	0	1
Malignancy	2 (lymphoma, brain tumour)	25 (lung cancer)	0

Data are presented as n or mean±sem. COVID-19: coronavirus disease 2019. ^#^: all non-COVID-19 samples were obtained from distal lung tissue to resected lung carcinoma (lobectomy or pneumonectomy); ^¶^: all non-COVID-19 acute pneumonia samples were from patients with aspiration pneumonia; ^+^: includes ischaemic heart disease, hypertension, cardiomyopathy, pulmonary embolism, peripheral artery disease and stroke.

### Cells and SARS-CoV-2 culture

SARS-CoV-2 (isolate 30–287, B.1.222 and B.1 strain (accession No MT459880.1)) was obtained through culture in Vero E6 cells (ATCC CRL-1586), from two different infected (COVID-19) patients. The virus was recovered from a nasopharyngeal swab, rinsed in 1 mL saline and filtered twice through a 0.22 nm filter. Virus stock was prepared by infecting fully confluent Vero-E6 cells in DMEM, 10% fetal bovine serum with antibiotics, at 37°C and 5% carbon dioxide. Virus stock was collected 4 days after inoculation, sequenced using next-generation sequencing (supplementary material) and the supernatant was frozen (−80°C) until use. Infections were carried out in 24-well plates, using SARS-CoV-2 at a 0.01 multiplicity of infection. Cells were either fixed with 4% paraformaldehyde or lysed with NucleoZOL (Macherey-Nagel) 17 days post-infection. Administration of the specific ataxia-telangiectasia-mutated (ATM) protein kinase inhibitor KU-55933 was carried out as described elsewhere [31]. Three independent experiments were performed. All manipulations were carried out in a biosafety level 3 facility.

### SARS-CoV-2 primary human lung alveosphere system

Formalin-fixed and paraffin-embedded sections from a three-dimensional alveosphere system infected with SARS-CoV-2 and corresponding controls (noninfected) were analysed. The alveospheres consist of self-renewing alveolar type 2 (AT2) cells that express the well-established AT2 cell markers HTII-280 and surfactant protein C, as well as of some cells expressing the AT1 cell marker HTI-56 [32]. Alveospheres were gently opened using pipetting to allow infection and to avoid cellular stress [32].

### Anti-SARS-CoV-2 (G2) antibody generation

Mouse immunisation and antibody collection, selection and specificity determination are described in the supplementary material. Transcriptome analysis of hybridomas and amino acid determination of selected clones are also provided. Four clones (479-S1, 480-S2, 481-S3 and 482-S4) are under patent application (V.G. Gorgoulis, D. Vassilakos, N. Kastrinakis. GR-patent application: 22–0003846810).

### RNA extraction and RT-qPCR detection

RNA extraction and RT-qPCR detection were performed as described previously (supplementary material) [33].

### Next-generation sequencing

Next-generation sequencing was performed as described previously (supplementary material) [34].

### Protein extraction and immunoblot analysis

Protein extraction and immunoblot analysis were performed as described elsewhere [31]. For details, refer to the supplementary material. Horseradish peroxidase conjugated anti-goat, anti-mouse and anti-rabbit secondary antibodies (1:1000 dilution) (Cell Signaling) were used. Primary antibodies were anti-APOBEC (apolipoprotein B mRNA editing enzyme, catalytic polypeptide-like) 3G/A3G (Abcam ab109727 and ab172694), anti-APOBEC3H (LSBio LS-C151868) and anti-glyceraldehyde 3-phosphate dehydrogenase (Cell Signaling).

### Immunocytochemistry and immunohistochemistry

Immunocytochemistry and immunohistochemistry were performed according to published protocols [34]. The following primary antibodies were applied overnight at 4°C: anti-SARS-CoV-2 (G2) monoclonal antibody (at a dilution 1:300), anti-SARS-CoV-2 monoclonal antibody (1A9 clone; Genetek), anti-angiotensin-converting enzyme (ACE)-2 (Abcam), anti-thyroid transcription factor (TTF)-1 (Dako), anti-surfactant protein (SP)-B (1B9 clone; Zeta Corporation), anti-p16^INK4A^ (Santa Cruz), anti-IL-1β (Abcam), anti-IL-6 (R&D systems), anti-phospho-histone (Ser^139^) H2AX (γΗ2ΑΧ) (Cell Signaling), anti-Ki67 (Abcam) and anti-p21^Waf1/Cip1^(Cell Signaling).

### Senescence-SenTraGor (GL13) methodology

SenTraGor (trademark of GL13 compound) staining and double-staining experiments were performed and evaluated as described previously [27, 35]. The mean percentage of SenTraGor-positive alveolar cells in ≥10 high-power fields (×400) per patient was quantified.

### Electron microscopy

Representative area from haematoxylin and eosin-stained paraffin sections of the lung autopsies of COVID-19 patients and non-COVID-19 controls were chosen under the light microscope and marked. Paraffin-embedded tissue was deparaffinised, rehydrated and fixed in 2.5% glutaraldehyde in PBS for 24 h and post-fixed in 1% aqueous osmium.

### Bioinformatic analysis for identification of mutational signatures in the SARS-CoV-2 genome

As described in detail in the supplementary material, candidacy for APOBEC deamination of C→U mutational substitutions in the SARS-CoV-2 genome of strains available in the GISAID database [36, 37] and in those obtained after cell culture infection, was examined and verified against experimentally validated APOBEC motifs (supplementary figure S7).

### Statistical analysis

Data are presented as mean±sd. Comparisons were performed with unpaired nonparametric Mann–Whitney U-test (comparison between two groups) or Kruskal–Wallis test followed by Dunn's *post hoc* analysis (comparison between three groups). The Wilcoxon paired nonparametric test was used to compare values of infected cells *in vitro*.

## Results

### Detection of SARS-CoV-2 in lung cells

**FIGURE 1:**
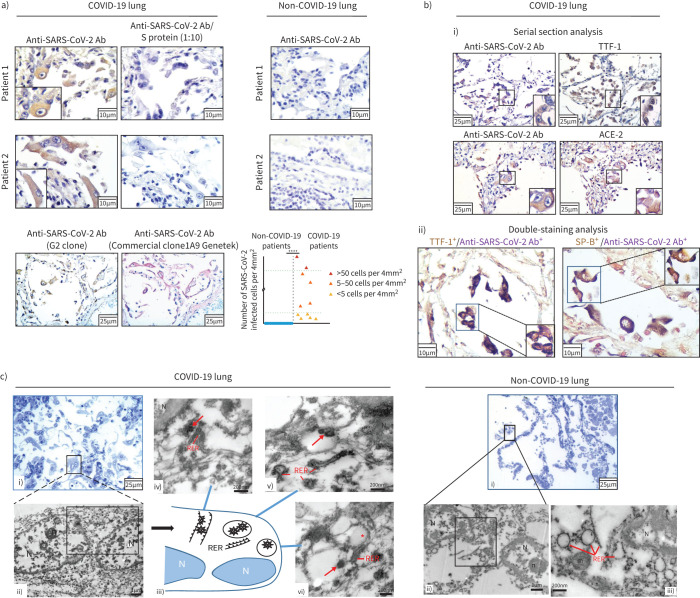
Detection of severe acute respiratory syndrome coronavirus 2 (SARS-CoV-2) in lung cells from patients who died of coronavirus disease 2019 (COVID-19). a) Representative images of SARS-CoV-2 (G2 mAb) immunohistochemistry staining in COVID-19 autopsy lung tissue. Competition with anti-peptide (S protein) showing specificity of the G2 immunostaining. Verification of G2 immunopositivity with a commercial anti-S antibody (Ab) (clone1A9 clone; Genetek). Representative negative control immunostaining in non-COVID-19 lung tissues. Graph shows quantification of SARS-CoV-2 staining in the lung samples. b) Detection of SARS-CoV-2 in alveolar type 2 (AT2) cells (confirmed by positive thyroid transcription factor (TTF)-1 and surfactant protein (SP)-B staining) and in angiotensin-converting enzyme (ACE)2-expressing cells (i); ii) double immunostaining is shown for SARS-CoV-2 and TTF-1. c) Detection of SARS-CoV-2 by transmission electron microscopy (EM) in a representative COVID-19 patient. Presence of SARS-CoV-2 i, ii) within AT2 cells and of iii, iv) virions in the proximity of the endoplasmic reticulum, iii, v, vi) as well as in cytoplasmic vesicles. ****: p<0.0001.

In order to detect SARS-CoV-2 in lung tissue we developed monoclonal antibodies which react against the receptor-binding domain of the spike protein of SARS-CoV-2 and identified a high-affinity antibody (G2), the validity of which was recently verified [38] (supplementary figure S1, supplementary table S1). SARS-CoV-2 was detected (using both our in-house G2 clone and a commercially available antibody from GeneTek) predominantly in AT2 cells, which were identified by TTF-1 and SP-B positivity, and in sparse inflammatory cells (alveolar and tissue macrophages) in all COVID-19 patients, ranging from <5 cells per 4 mm^2^ tissue to >50 cells per 4 mm^2^ tissue (figure 1a, supplementary table S1a). Surface epithelial cells in small peripheral airways stained also positive in certain cases (supplementary figure S2). SARS-CoV-2-infected AT2 cells were occasionally large and appeared isolated (denuded or syncytial) or clustered (hyperplasia), exhibiting a variety of topological distribution (figure 1a). These cells co-expressed the ACE2 receptor (figure 1b), supporting SARS-CoV-2 infection being mediated by the ACE2 receptor [39]. In addition, electron microscopy analysis in representative COVID-19 cases confirmed the presence of virus within AT2 cells (figure 1c) and high magnification revealed virions in the proximity of the endoplasmic reticulum, indicating their likely assembly and budding, as well as virions residing in cytoplasmic vesicles, implying their transfer and release into the extracellular space.

### Senescence in SARS-CoV-2-infected cells

**FIGURE 2:**
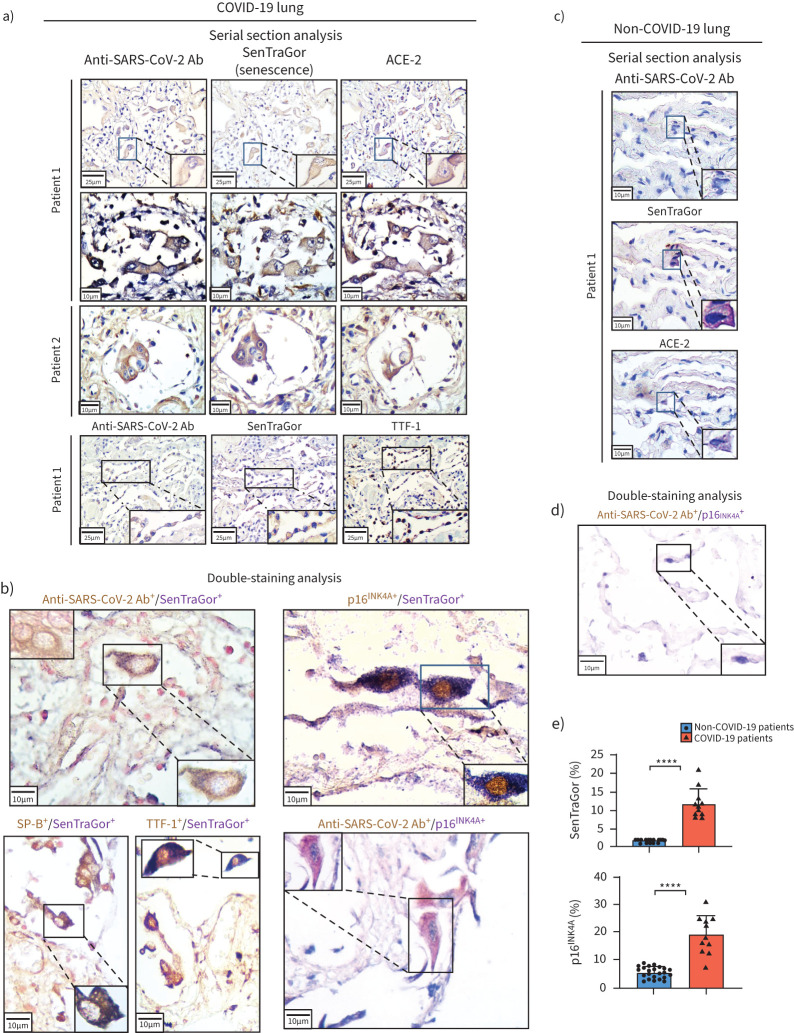
Senescence in severe acute respiratory syndrome coronavirus 2 (SARS-CoV-2)-infected cells. a) Representative images of SARS-CoV-2, SenTraGor (senescence) and angiotensin-converting enzyme (ACE)2 staining in serial sections of coronavirus disease 2019 (COVID-19) lung tissue. b) Double-immunostaining analysis for SARS-CoV-2, SenTraGor (senescence), p16^INK4A^, thyroid transcription factor (TTF)-1 and surfactant protein (SP)-B in COVID-19 lung tissue. c) Representative results from serial staining for SARS-CoV-2, SenTraGor (senescence) and ACE2, and d) double-staining for SARS-CoV-2 and p16^INK4A^ in non-COVID-19 lung tissue. e) Graphs showing increased expression of SenTraGor and p16^INK4A^ in COVID-19 lung tissue. Ab: antibody. ****: p<0.0001.

A proportion of SARS-CoV-2-infected AT2 cells (range 8–21%) displayed a senescent phenotype, with positive staining for SenTraGor and p16^INK4A^ (figure 2a–c, supplementary figure S3) [12, 29, 30]. SenTraGor is an established marker of senescence and has the unique property of detecting senescent cells in any setting, including archival material (formalin-fixed paraffin-embedded tissue) [12, 27, 35]. By contrast, lung tissues exhibiting fibrosis, atelectasis and inflammatory infiltrates from age-matched non-COVID-19 cases with similar comorbidities (table 1, supplementary table S1), including a separate control cohort of patients with acute pneumonia (aspiration pneumonia), showed significantly lower senescence (range 1–2%, p<0.0001) (figure 2a–c, supplementary figure S4), suggesting that SARS-CoV-2 infection may induce senescence.

**FIGURE 3:**
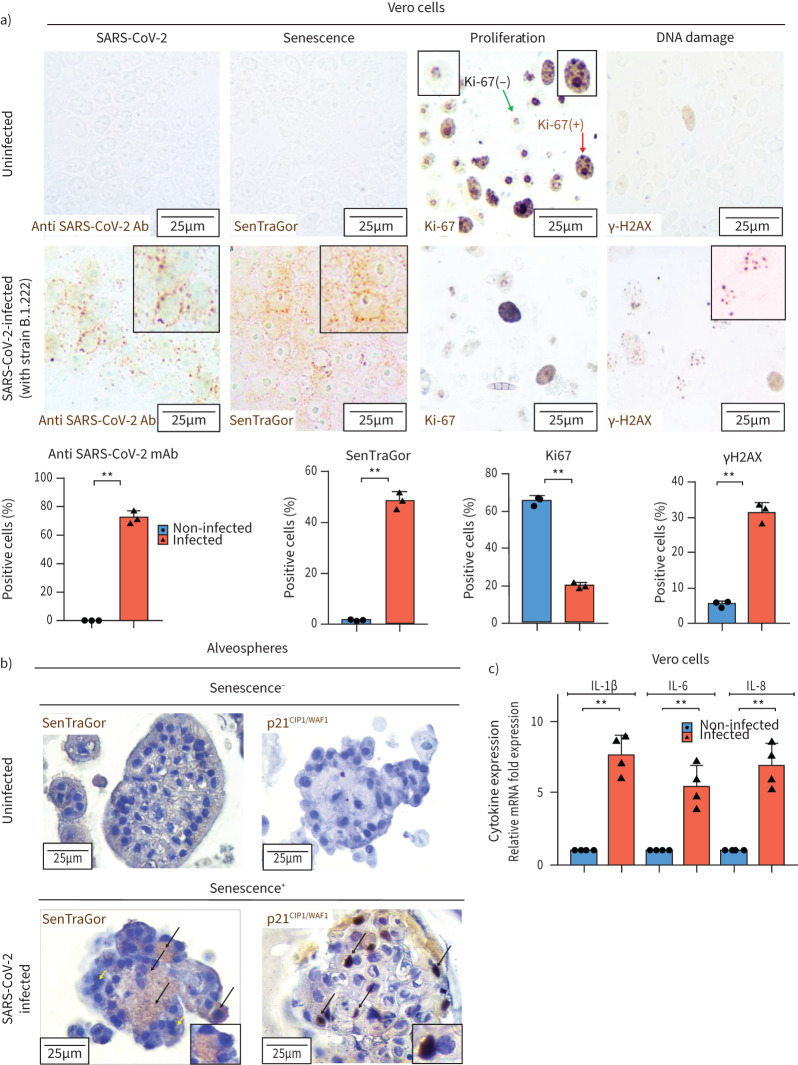
Severe acute respiratory syndrome coronavirus 2 (SARS-CoV-2)-induced senescence in Vero-E6 cells and alveospheres. a) SARS-CoV-2 presence, senescence induction, cellular proliferation and DNA damage activation, with corresponding quantitative histograms, in Vero-E6 cells with and without SARS-CoV-2 infection (17 days postinfection). b) Senescence induction (assessed by SenTraGor and p21^WAF1/CIP1^ staining) in alveolar cells (alveospheres) of a primary lung alveolar three-dimensional model following SARS-CoV-2 infection (black arrows) in comparison to nonsenescent ones (yellow arrows). Absence of senescence is clearly evident in noninfected alveolar cells. c) Graph showing induction of senescence-associated secretory phenotype-related cytokines following SARS-CoV-2 infection of Vero-E6 cells (mRNA expression normalised against β_2_-microglobulin mRNA expression). Ab: antibody; IL: interleukin. **: p<0.01.

In order to demonstrate that SARS-CoV-2 can induce cellular senescence, we infected Vero cells with a viral strain isolated from a COVID-19 patient. Vero-E6 cells provide an established cellular system for viral propagation and studies, as apart from their high infectivity to SARS-CoV-2 they are among the few cell lines demonstrating SARS-CoV-2-mediated cytopathic effects, an essential aspect in diagnostics [40, 41]. Infection was carried out at a low multiplicity of infection to mimic natural coronavirus infection [42]. In line with our hypothesis, the infected cells following an initial surge of cell death reached an equilibrium demonstrating clear evidence of senescence (increased SenTraGor positivity, increased p21^WAF1/CIP1^ and reduced Ki-67), as compared to the noninfected control cells at 17 days post-infection (figure 3, supplementary figure S5a). As Vero cells lack p16^INK4A^ [43], the most likely trigger of senescence is DNA damage, as reported previously [12, 13]. DNA damage measured by γ-H2AX immunostaining was evident in SARS-CoV-2 infected cells (figure 3a). Treatment of infected cells with a selective ATM inhibitor (KU-55933) dramatically decreased senescence assessed by SenTraGor staining (supplementary figure S6). ATM has been reported previously as a key driver of NF-κB-dependent DNA-damage-induced senescence [44]. It appears that genotoxic stress results from a vicious cycle imposed by the virus in host cells as it hijacks most intracellular protein machineries [11, 45]. Likewise, cellular senescence was identified in infected alveolar cells/alveospheres of an established primary lung alveolar three-dimensional model, confirming the *in vivo* observations and the findings in Vero cells (figure 3b, supplementary figure S5b).

### Senescence-associated secretory phenotype

**FIGURE 4:**
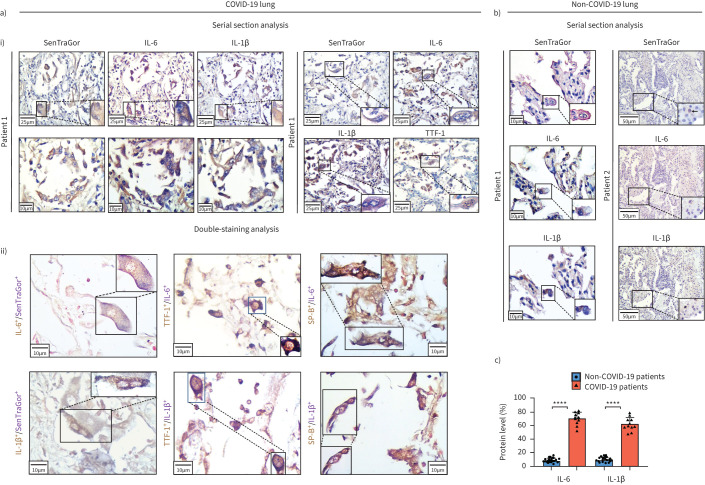
Senescence-associated secretory phenotype in coronavirus disease 2019 (COVID-19) lung tissues. a) Representative staining results (at low and high magnification) of SenTraGor, interleukin (IL)-6, IL-1β and thyroid transcription factor (TTF)-1 and/or surfactant protein (SP)-B in i) corresponding serial sections and ii) as double-immunostaining analysis of COVID-19 lung tissue. Original magnification: ×400. b) Representative staining results showing absence or minimal levels of SenTraGor, IL-6 and IL-1β in age-matched non-COVID-19 control samples. c) Corresponding graphs depicting quantification of IL-6 AND IL-1β in COVID-19 and non-COVID-19 patients. ****: p<0.0001.

We found very high expression of both IL-1β and IL-6 by senescent AT2 cells in the lungs of COVID-19 patients, while in the non-COVID-19 control cases, including samples from patients with acute pneumonia, expression was very low in the few senescent AT2 cells detected in lung tissue with fibrosis, atelectasis and inflammatory infiltrates (p<0.0001) (figure 4a–c, supplementary table S1). As both cytokines are key components of the inflammatory response to SARS-CoV-2, our findings imply that this pattern of inflammation may be due to the SASP as a result of cellular senescence in COVID-19 patients. Likewise, SARS-CoV-2 senescent Vero cells displayed expression of SASP-related cytokines, as assessed by our algorithmic assessment of senescence, supporting our *in vivo* findings (figure 3c).

### SARS-CoV-2 extrapulmonary dissemination

**FIGURE 5:**
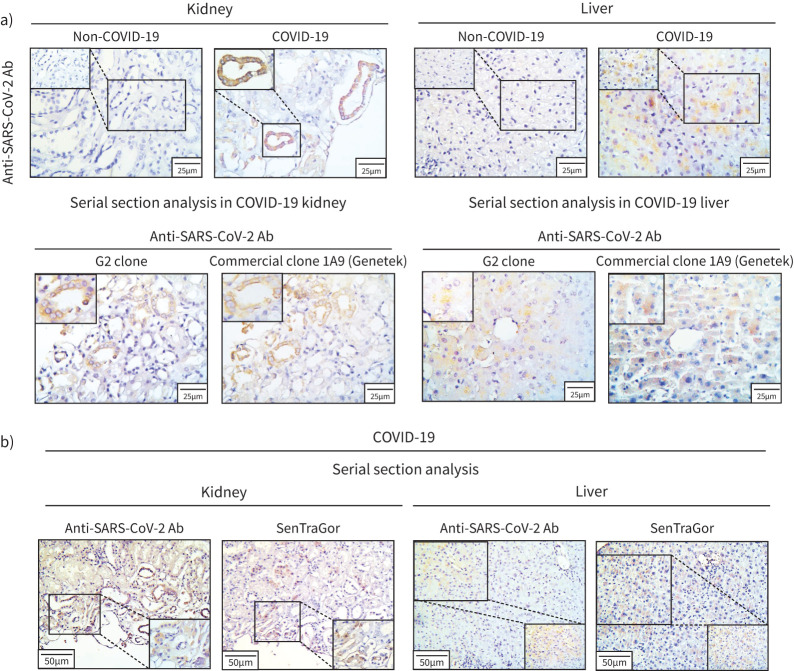
Severe acute respiratory syndrome coronavirus 2 (SARS-CoV-2) dissemination in extrapulmonary sites. a) Representative staining results of SARS-CoV-2 in the kidney and the liver of a coronavirus disease 2019 (COVID-19) patient. Absence of the immunohistochemical signal is evident in the corresponding tissues of a representative non-COVID-19 case. b) Serial section analysis of the representative COVID-19 case (see a) depicting concurrent positivity for SARS-CoV-2 and the senescence marker (SenTraGor) in cells of the liver and kidney. Ab: antibody.

SARS-CoV-2 immunoreactivity was additionally detected by applying both our in-house G2 clone and a commercial one from GeneTek in serial sections of available kidney and liver tissue from one COVID-19 patient (figure 5a). A clear cytoplasmic signal was evident in a number of renal tubules as well as in areas of the liver parenchyma. In contrast, in tissues from non-COVID-19 cases the signal was absent, as expected (figure 5a). Interestingly, by serial section analysis, areas harbouring the virus also exhibited senescence (SenTraGor positivity), implying occurrence of SARS-CoV-2-induced senescence in extrapulmonary sites (figure 5b).

### Infected senescent cells and SARS-CoV-2 mutagenesis

Recently, we hypothesised that senescent cells could represent a source of SARS-CoV-2 quasispecies generation [46]. This assumption was based on the fact that the apoptotic tolerant nature of senescent cells allows the virus to be hosted for longer periods compared to other cells with higher cell turnover [47, 48], thus rendering the SARS-CoV-2 virome more susceptible to host-mediated editing. APOBEC enzymes are well known to participate in viral genome editing [12, 33, 49]. Notably, we showed these enzymes to be highly expressed in senescent cells [49].

**FIGURE 6:**
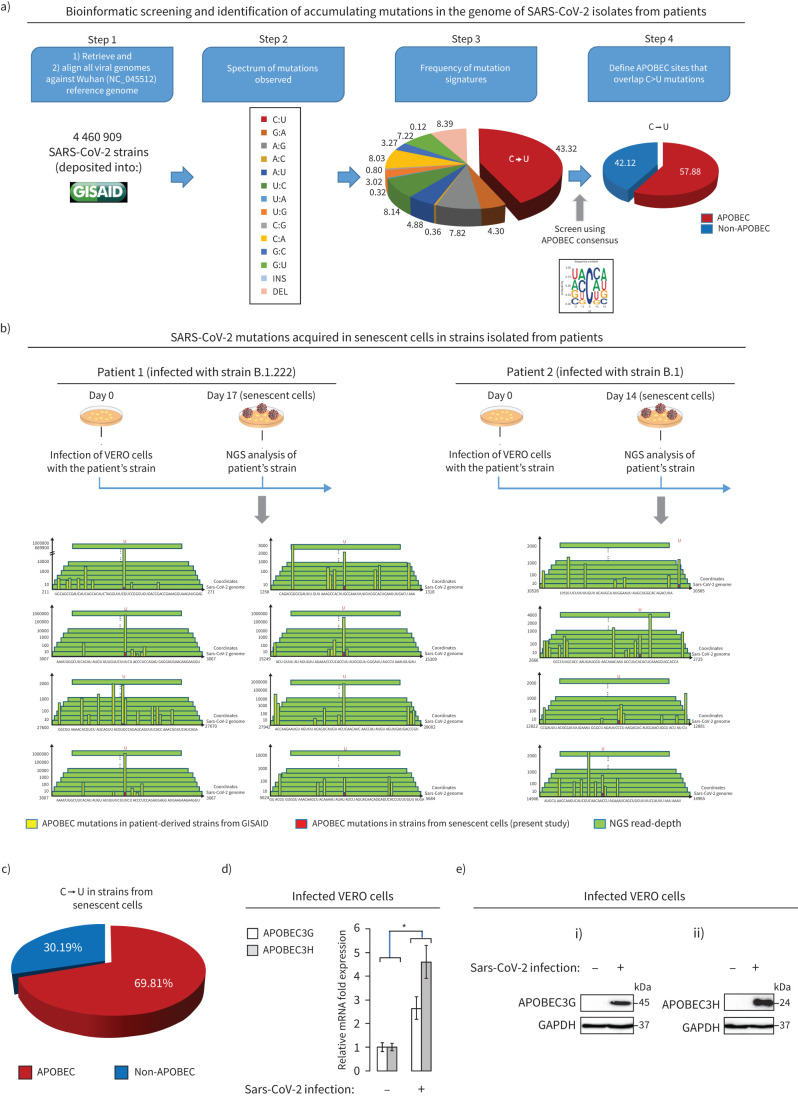
Infected senescent cells as a putative source for severe acute respiratory syndrome coronavirus 2 (SARS-CoV-2) quasispecies variant generation. a) Schematic layout presenting the identification of apolipoprotein B mRNA-editing (APOBEC)-mediated mutations in the genome of 4 672 296 SARS-CoV-2 strains available in GISAID database. For algorithmic assessment see supplementary figure S7 and the supplementary materials and methods. b) Identification of SARS-CoV-2 mutations acquired in senescent cells in two strains (B.1.222 and B.1) isolated from two different patients, following cell-culture infection for the time periods indicated (day 1 and day 14). Yellow bars show the frequency of C→U substitutions observed in the GISAID database in the next-generation sequencing (NGS) read counts (green pileups) and red bars depict the C→U frequency of mutation observed post-infection of cells in the current study, in the genome of the two employed SARS-CoV-2 strains. These sites are ranked as highest relatively to the C→U counts as observed from the GISAID database. The genomic sequences flanking each C→U are also presented, while their coordinates are shown relative to the superimposed SARS-CoV-2 genome. NGS reads were confirmed in triplicate. c) Higher frequency of C→U substitutions within APOBEC consensus (supplementary figure S7) in the genome of isolated viral progenies obtained in Vero-E6 cells. d) Increased APOBEC3G and APOBEC3H mRNA levels in infected Vero-E6 cells. e) Increased APOBEC3G and APOBEC3H protein levels in infected Vero-E6 cells.

Updating our initial bioinformatic investigation [46], we screened ∼4 500 000 SARS-CoV-2 strains from the GISAID database [36, 37]. This analysis mostly indicated that the predominant signature of SARS-CoV-2 variants is APOBEC-mediated (figure 6), in line with recent literature [50]. To validate our hypothesis, we proceeded to cell-culture infection with two strains isolated from patients and obtained progenies from senescent cells (figure 6b). Next-generation sequencing analysis demonstrated that the collected viral genomes acquired mutations. Importantly, the predominant pattern was APOBEC-driven, in agreement with the bioinformatic analysis of the GISAID database. Moreover, and in line with our presumption and our previous study [49], the SARS-CoV-2-induced senescent cells demonstrated significantly higher mRNA and protein levels of the APOBEC enzymes, particularly G and H (RNA editing cytoplasmic variants), which are reported to play a pivotal role in viral RNA editing [12, 33, 49]. Other mutation signatures were also found both in the cell-culture-isolated virome genomes and in the strains from GISAID, and maybe the outcome of the oxidative stress present in the senescent cells [51].

## Discussion

We have demonstrated the presence of SARS-CoV-2 in AT2 cells of patients who died from COVID-19 using our novel and a commercial anti-SARS-CoV-2 antibody against its spike protein and by electron microscopy. We have shown for the first time following the senescence-detecting algorithm *in vivo* that a proportion of SARS-CoV-2-infected AT2 cells exhibit features of cellular senescence (as demonstrated by significantly increased staining with the novel senescence marker SenTraGor and also of p16^INK4A^) [12, 27, 35]. In this respect, our investigation encompassing serial section analysis and costaining is the first to demonstrate *in vivo* which cells are truly senescent and infected. The finding that in age-matched non-COVID-19 controls, including lung tissue samples from a cohort of patients with acute pneumonia, the percentage of senescent cells was much lower (1–2%) than that of the COVID-19 patients (8–21%) strongly indicates that SARS-CoV-2 triggers senescence. To confirm that SARS-CoV-2 induces cellular senescence *per se*, we demonstrated that infection of epithelial cells with SARS-CoV-2 virus (B.1.222 strain) *in vitro* increased SenTraGor staining and induced DNA damage, measured by increased γ-H2AX expression. Importantly, we found that inhibition of ATM, an apical orchestrator of the DNA damage response (DDR) pathway, dramatically reduced senescence in infected samples, suggesting SARS-CoV-2-induced senescence being mediated by DNA damage and activation of the DDR pathway. In line with our *in vivo* and *in vitro* findings, SARS-CoV-2-induced senescence was identified in a previously established primary lung alveolar three-dimensional model [32]. The fact that alveospheres underwent gentle mechanical manipulations is unlikely to have contributed to the occurrence of senescence, given that such interventions are frequently applied on three-dimensional organoid systems and no senescence phenotypes have been reported nor are evident from the post-infection proliferation rates and culture [52].

In addition, we demonstrated that cells infected with SARS-CoV-2 exhibit high expression of IL-1β and IL-6, both components of the SASP and implicated in systemic features of severe COVID-19 [3, 4]. Therefore, our *in vitro* and *in vivo* findings suggest that SARS-CoV-2 attaches to AT2 cells *via* ACE2 to infect these cells, and through activation of DDR signalling roots, induces cellular senescence and associated proinflammatory phenotype (SASP). In line with our observations, Lee *et al.* [29] demonstrated that SARS-CoV-2-induced senescence exhibited enhanced γ-H2AX DNA damage foci that were abrogated by the reactive oxygen species (ROS) scavenger N-acetylcysteine, suggesting the implication of ROS and oxidative stress (supplementary figure S8). Moreover, infected cells displayed SASP-positive senescence mediated by p53 and activation of the cGAS/STING signalling pathway [29]. They also demonstrated that senolytics reduced COVID-19 lung disease and inflammation in infected hamsters and mice [29], further suggesting SASP as an outcome of virus-induced senescence. Camell *et al.* [53] reported that treatment with senolytics of old mice infected with a SARS-CoV-2-related mouse β-coronavirus reduced inflammation. Based on our findings and recent evidence, a potential mechanistic scenario could be as follows (supplementary figure S8). Upon entrance, the virus highjacks certain energy-consuming functions related to RNA processing, translation and the endoplasmic reticulum [11]. Thus, an energy shift occurs, resulting in increased ROS production, oxidative stress and DNA damage/DDR pathway activation. In addition, DDR and the subsequent cell cycle arrest may be driven by an interaction of coronavirus nsp13 protein and DNA polymerase δ [54] and SASP *via* the cGAS/STING and other DNA-damage-dependent pathways (supplementary figure S8). Therefore, our prior work showing that DNA damage/DDR is a potent inducer of senescence signifies its importance in the establishment of the virus-induced senescence phenotype [31].

Senescent cells are in a state of cell cycle arrest, but remain metabolically active and secrete a typical profile of inflammatory proteins known as the SASP. SASP components include the proinflammatory cytokines IL-1β and IL-6, which are elevated in plasma of COVID-19 patients that have acute respiratory distress syndrome or systemic inflammatory features. We demonstrate that in COVID-19, senescence alters the properties and function of AT2 cells, producing cytokines that could be released into the systemic circulation, amplifying and perpetuating chronic inflammation. Of course, the availability of tissue samples and the absence of more refined COVID-19 *in vitro* and *in vivo* models make it difficult to draw robust conclusions. It is likely that SARS-CoV-2 spreads from epithelial cells in the lower airways to infect AT2 cells in the alveolar walls, which express ACE2, and causes local senescence and inflammation *via* SASP in the lung. In line with this notion, it has been previously reported *in vitro* that cellular senescence has also a role as a natural antiviral defence mechanism and that SASP acts as the major contributor of this response, activating and recruiting the immune system to clear out the infection [21, 22]. Subsequently, other cellular compartments (stem/progenitor, endothelial and inflammatory cells) may be affected *via* different cell- and noncell-autonomous mechanisms, leading to aberrant immune responses, chronic inflammation, tissue dysfunction and/or fibrosis, and eventually to lung damage and failure [26, 55]. In particular, senescence in stem/progenitor cells impairs lung regenerative capacity. Immunosenescence results in elevated neutrophils-to-lymphocytes ratio and high IL-6 production. In addition, IL-1, IL-6, CXCL8 and TNF-α might be crucial for maintaining chronic inflammation, and TGF-β, plasminogen activator inhibitor-1 and matrix metalloproteinases might favour fibrosis *via* the expression of fibrotic genes (ACTA2, COL1A1, COL1A2 and FN1) in the surrounding microenvironment. The virus may then enter the circulation and senescence may subsequently spread systemically to affect other organs (figure 5), leading to multiorgan failure/multimorbidity and death in the acute phase [1, 25], or leading to post-acute sequelae of COVID-19 (PASC or long COVID), an evolving syndrome with long-term complications [26, 56]. In this manner, senescence could also predispose to disease severity upon re-infection by SARS-CoV-2 or infection by other viruses and even negatively impact vaccine efficacy, similarly to that proposed for aged individuals [26].

An additional implication relates to the prolonged survival of senescent cells that are infected with the virus, as senescent cells are resistant to apoptosis and clearance by efferocytosis [12, 14]. Such a context can provide an extended time “window” for virus replication, therefore exposing its genome to host-mediated editing. Along this vein we showed that the senescent cell compartment acts as a “fertile” environment for mutational evolution of the virus, as it is more susceptible to APOBEC-3G and 3H RNA editing (figure 6) [33, 49]. Of course, other mechanisms extending survival and replication of SARS-CoV-2 may take place. Coronaviruses code for an important multifunctional enzyme termed papain-like protease which exerts intrinsic deubiquitinating and deISGylating activities. The latter is related to interferon-stimulated genes (ISG) upregulation and can serve to antagonise the host's immune response which would otherwise hinder infection [57]. In this manner, increased and prolonged viral replication and exposure of its genome to host-mediated editing and quasispecies generation could also occur. Taking into account that interferons are known senescence inducers, the picture gets even more complicated, rendering it difficult to discriminate ISG-mediated mechanisms from those involving virally induced senescence [58]. Additional studies are needed and the guideline algorithmic approach for *in vivo* senescence assessment, followed in the current work, is anticipated to clarify in the future a lot of unanswered questions in clinical settings [12, 27]. Notably, in the recently identified Omicron (B.1.1.529) SARS-CoV-2 variant/strain the APOBEC signature was identified as the predominant mutational profile (supplementary figure S9). To better understand the role of APOBEC in the generation of viral mutations, inhibition of APOBEC proteins by RNA interference would be valuable. However, this is very challenging, since APOBEC suppression induces DNA damage and sensitises cells to stress-induced death [59–61]. Interestingly, and in line with our concept linking cellular senescence with viral mutagenesis, by conducting a bioinformatic analysis, we found that viruses bypassing cellular senescence (oncogenic) exhibit a significant lower mutation burden compared to viruses inducing senescence (supplementary figure S10). Given that mutations can affect the effectiveness of vaccines, our findings could imply a potential implication of virus-induced senescence in vaccination strategies. Further studies are required.

Despite the fact that up to the present this is the largest clinical study demonstrating virally induced senescence by SARS-CoV-2, a limitation of our study is the relatively small sample size of the COVID-19 lung autopsies, due to the difficulty of accessing this rare material, given that autopsies are limited and were performed only in the initial phase of the COVID-19 outbreak in order to identify the pathological basis of this new entity. Another limitation due to the nature of the disease is that pathological features, such as cellular senescence in lungs, can only be investigated in cadaverous material, which represents the most severe outcome of the spectrum of COVID-19 clinical manifestations. Therefore, evaluation of senescence in less-severe conditions is not currently feasible. Moreover, in our study we examined 18 cases of aspiration pneumonia, which displayed similar degrees of senescence to other noninfected control cases. It would be interesting to extend these studies in other types of pneumonia. However, material from patients with an acute, but not COVID-19-related inflammatory response is rare, given that these patients commonly recover following treatment and in the infrequent cases of fatal outcome autopsy is rarely performed.

Overall, SARS-CoV-2-induced senescence justifies the therapeutic application of senotherapeutics for the treatment of COVID-19 patients and possibly for long-COVID syndromes [56]. Quercetin was recently tested against standard care in two randomised clinical trials (ClinicalTrials.gov identifiers NCT04578158 and NCT04861298) in patients with confirmed SARS-CoV-2 infection and mild COVID-19-associated symptoms. In both trials, senolytic intervention led to clinical improvement [62, 63]. Overall, these data imply the beneficial role of senolytics in significantly improving the outcome during SARS-CoV-2 infection.

## Supplementary material

10.1183/13993003.02951-2021.Supp1 **Please note:** supplementary material is not edited by the Editorial Office, and is uploaded as it has been supplied by the author.
Supplementary material ERJ-02951-2021.Supplement

## Shareable PDF

10.1183/13993003.02951-2021.Shareable1 This one-page PDF can be shared freely online.
Shareable PDF ERJ-02951-2021.Shareable
