# Supplementary material for: Pulmonary infection by SARS-CoV-2 induces senescence accompanied by an inflammatory phenotype in severe COVID-19: possible implications for viral mutagenesis
Source: Eur Respir J. 2022 Aug 18;60(2):2102951. doi: 10.1183/13993003.02951-2021 (PMC8796696; doi:10.1183/13993003.02951-2021)
Supplement: Supplementary file 1 [file ERJ-02951-2021.Supplement.pdf]

ONLINE DATA SUPPLEMENT

Material and Methods

RNA extraction and Reverse-Transcription real-time PCR (RT-qPCR) detection

SASP cytokine mRNA analysis

RNA was extracted using the Nucleospin RNA kit (Macherey-Nagel #740955) according to the manufacturer's instructions. 1 μg RNA was used for cDNA preparation with Primescript^{TM} RT Reagent Kit (Takara #RR037A). RT-qPCR was performed utilizing SYBR Select Master Mix (Life technologies #4472908) on a DNA-Engine-Opticon (MJ-Research) thermal cycler. Primer sequences employed were: IL-1β Fw: 5'-GGAAGACAAATTGCATGG-3', Rv: 5'-CCCAACTGGTACATCAGCAC-3'; IL-6 Fw: 5'- AGAGGCACTGGCAGAAAAC-3', Rv: 5'-TGCAGGAACTGGATCAGGAC-3'; IL-8 Fw: 5'-AGGACAAGAGCCAGGAAGAA-3', Rv: 5'-ACTGCACCTTCACACAGAGC-3'; B2M: β2-microglobulin (reference) gene Fw: 5'-TCTCTGGCTGGATTGGTATCT-3', Rv: 5'-CAGAATAGGCTGCTGTTCCTATC-3' [1]. Results, averaged from three independent experiments, are presented as n-fold changes after Sars-CoV-2 infection relatively to the non-infected condition, using the 2-ΔΔCT method.

Viral RNA detection

RNA was extracted using the NucleoSpin Virus RNA purification kit (Macherey-Nagel #740.983) according to the manufacturer's instructions. RT-qPCR was performed utilizing the One Step PrimeScript III RT-PCR Kit (Takara # RR601B) on a Rotor-Gene Q 6000 (Qiagen) thermal cycler following the manufacturer's instructions and using the CDC N-

gene directed primers [**https://www.cdc.gov/coronavirus/2019-ncov/lab/rt-pcr-panel-primer-probes.html**].

Next Generation Sequencing (NGS)

The Ion AmpliSeq Library Kit Plus was used to generate libraries following the manufacturer's instruction, employing the Ion AmpliSeq SARS-CoV-2 RNA custom primers panel (ID: 05280253, Thermo Fisher Scientific). Briefly, library preparation steps involved reverse transcription of RNA using the SuperScript VILO cDNA synthesis kit (Thermo Fisher Scientific), 17-19 cycles of PCR amplification, adapter ligation, library purification using the Agencourt_AMPure XP (Beckman Coulter), and library quantification using Qubit Fluorometer high-sensitivity kit. Ion 530 Chips were prepared using Ion Chef and NGS reactions were run on an Ion GeneStudio S5, ion torrent sequencer (Thermo Fisher Scientific). Samples were run in triplicates.

Protein extraction and immunoblot analysis

Total protein extracts were obtained by resuspension in 50 mM Tris/HCl pH 8.0, 150 mM NaCl, 0,1% SDS, 0,5% sodium deoxycholate, 1% NP-40 adjusted with protease and phosphatase inhibitors. Lysate was centrifuged at 13,400 rpm at 4°C for 15 min. The supernatant was collected and proteins quantified using Protein assay dye concentrate (BIO-RAD). Thirty micrograms of protein were adjusted with Laemmli buffer (Merck, 38733) and loaded on acrylamide/bis-acrylamide gels. Gel electrophoresis was followed by transfer to PVDF membrane (Macherey-Nagel, 741260), while signal development was carried out by Clarity Western ECL Substrate (Bio-Rad, 1705060) chemiluminescence and captured on an iBright CL750 Imaging System (Thermo Fisher Scientific). Horse Radish

Peroxidase conjugated anti-goat, anti-mouse and anti-rabbit secondary antibodies (1:1000 dilution) (Cell Signaling) were used. Primary antibodies were: anti-APOBEC3G/A3G (Abcam ab109727 and ab172694), anti-APOBEC3H (LSBio LS-C151868) and anti-GAPDH (rabbit, Cell Signaling, 2118S, 1:2000).

Anti-SARS-CoV-2 antibodies

Generation

A series of monoclonal antibodies against SARS-CoV2 Receptor Binding Domain (RBD) of spike protein were produced according to a modified method of Koehler and Milstein (Koehler and Milstein, 1975). Briefly, twelve BALB/c mice of 5 weeks of age were immunized intraperitoneally (i.p.) with 25μg of SARS-Cov2 protein (Trenzyme GmbH, Germany). All immunization and animal handling were in accordance with animal care guidelines as specified in EU Directive 2010/63/EU. After 5 cycles of immunization, mice were sacrificed, spleenocytes were collected and fused with P3X63Ag8.653 (ATCC® CRL1580™) following a modified method of Koehler and Milstein. Positive clones and antibody specificity were determined through extensive immunosorbent assays. Four clones, namely 479-S1, 480-S2, 481-S3 and 482-S4 are under patent application (Gorgoulis VG, Vassilakos D and Kastrinakis N *(2020)* GR patent application no: 22-0003846810).

RNA sequence determination and amino acid prediction

RNA was collected from biological duplicates of generated hybridomas as described elsewhere [2]. RNA samples were processed according to manufacturer's instructions, using the following kits: NEBNext® Poly(A) mRNA Magnetic Isolation Module (E7490S),

NEBNext® Multiplex Oligos for Illumina® (Index Primers Set 1, NEB7335) and NEBNext® Ultra™ II Directional RNA Library Prep with Sample Purification Beads (E7765S). After successful QC (RNA 6000 Nano bioanalyzer, Agilent) and quantity measurements (Qubit™ RNA HS Assay Kit, ThermoFisher), 1ug was used for mRNA selection, cDNA construction, adaptor ligation and PCR amplification (11 cycles), according to the manufacturer's protocol: (https://international.neb.com/products/e7760-nebnext-ultra-ii-directional-rna-library-prep-kit-for-illumina#Product%20Information). The 479-G2-ATCACG index from NEB E7335 was used. The final libraries were analyzed with Agilent High Sensitivity DNA Kit on an Agilent bioanalyzer, quantitated (Qubit dsDNA HS Assay Kit, Thermofisher) and, after multiplexing, were run using a NextSeq 500/550 Mid Output Kit v2.5 (150 cycles), paired end mode on a NextSeq550 (Illumina) at final concentration 1,3pM with 1% PhiX Control v3.

Fastq files were demultiplexed with Flexbar [3]. Quality control of the Fastq files was assessed with FastQC tools [4]. Adapter sequences were removed with Cutadapt program [5] with the following parameters: quality trimming was set to 20 and the minimum allowed nucleotide length after trimming was 20 nucleotides using --pair-filter=any to apply the filters to both paired reads. A two way alignment mode was followed to identify the antibody clone. More precisely alignments were performed with Bowtie2 [6] with parameters set as following: -D 20 -R 3 -N 1 -L 20 -i S,1,0.50 –no-mixed --no-discordant against an index made from IMGT database http://www.imgt.org/ having downloaded all mouse and human IG genes. Also this mode of alignments was executed for quality control and visualization of the aligned reads spanning the IG gene segments on the genome browser. The second mode refers to the determination and

reconstruction of the clones. This was performed with MiXCR suite [7]. At first, alignments against the IG repertoire were performed with kaligner and visualization of alignments was assessed. It was observed that the use of kaligner gave better results with higher clone hits regarding the VH and VL segments. Full assembly of the clones was performed. A full report of the number of reads and assembly of CDR and FR clones is provided in clones479_S1kalign.txt. The clones with the highest number of reads and coverage across the V, D, J segments were considered. The reported matched sequences were also checked with IgBlast tool https://www.ncbi.nlm.nih.gov/igblast/. In addition, after the assembly of the amino acid reconstruction of the FR and CDR regions of the full variable fragment for both the Heavy and Light antibody chains, a 3D visualization was also determined via folding the V protein fragment with iTassser suite [8]. The above analysis has been extensively described in Gorgoulis VG, Vassilakos D and Kastrinakis N. *(2020)* GR patent application no: 22-0003846810.

Immunocytochemistry (ICC)-Immunohistochemistry (IHC)

Method: ICC and IHC were performed according to previous published protocols [9]. In brief, 3 μm thick sections from formalin-fixed paraffin embedded (FFPE) lung tissues were employed. Antigen retrieval was heat-mediated in 10 mM citric acid (pH 6.0) for 15-20 minutes. The following primary antibodies were applied: i) the anti-SARS-CoV-2 (G2) monoclonal antibody (dilution 1:300), ii) anti-SARS-CoV-2 mononclonal antibody [1A9 clone Genetek, Cat.no: GTX632604 (dilution 1:100], iii) anti-ACE-2 [Rabbit polyclonal antibody Abcam, Cat.no: ab15348 (dilution 1:200)], iv) anti-TTF-1 [mouse monoclonal antibody Dako, Clone 8G7G3/1, Cat.no: M3575 (Ready-to-Use)], v) anti-Surfactant Protein B (SP-B) [1B9 clone ZETA Corporation Cat.no: Z2196ML (dilution 1: 100)], vi) anti-

p16^{INK4A} [mouse monoclonal antibody Santa Cruz, clone: F-12, Cat.no.:sc-1661. (dilution 1:100)], vii) IL-1β [Rabbit polyclonal antibody Abcam, Cat.no: ab2105 (dilution 1:150)], viii) IL-6 [mouse monoclonal antibody R&D systems, clone: Clone: 6708, Cat.no:MAB206 (dilution 1:100)], ix) anti-γH2AX [Phospho-Histone H2A.X (Ser139) clone:(20E3) Rabbit cat.no.:mAb #9718 dilution: 1/400] and x) anti-Ki67 [clone: SP6 cat.no.: ab16667 dilution: 1/250], anti- p21$^{WAF1/CiIP1}$ [clone: 12D1 Rabbit cat.no.: mAb #2947 dilution 1/100], all overnight at 4°C. Development of the signal was achieved using the Novolink Polymer Detection System (Cat.no: RE7150-K, Leica Biosystems). Specimens were counterstained with hematoxylin.

Negative Controls for the anti-SARS-CoV-2 (G2) monoclonal antibody: *i) Biological,* comprising previously published and new lung tissue samples from a cohort of 60 cases that underwent surgery prior to COVID-19 outbreak [10]. *ii) Technical:* a. Omission of the G2 primary monoclonal antibody, b. Blocking of the G2 primary monoclonal antibody using the corresponding S-protein (Cat.no. P2020-029, Trenzyme) in a 1:10 (G2/Spike protein) ratio and c. Two slides per case were employed for each staining or control experiment.

Evaluation of G2 staining: Cells were considered positive irrespective of the staining intensity. Two different semi-quantitative IHC evaluation approaches, previously described were adopted [11, 12] According to the first, the number of G2 positive cells per 4mm^2 was encountered and scored according to the following criteria: (+) for positive staining in <5 cells per 4 mm^2, (++) for positive staining in 5–50 cells per 4mm^2 and (+++) for positive staining in >50 cells per 4 mm^2 [10]. Regarding the second one, the number of G2 positive cells per whole slide was estimated and subsequent scores were assessed: (+) between one and five positive cells per whole slide (scattered cells), (++) more than

five cells per whole slide but no foci (isolated cells) and (+++) more than 10 cells in one × 20 field (with foci) [11]. For $p16^{INK4A,}$ the mean percentage of positive alveolar cells in at least 10 high power fields (x400) per patient was measured. This information has been included in the methods section (Main and Online Suppl Data). For IL-6 and IL-1b, the percentage of immunopositive cells was encountered [13]. Evaluations were performed blindly by four experienced pathologists (KE, PF, CK and VG) and intra-observer variability was minimal (p≤0.05).

Bioinformatic analysis for identification of mutational signatures in the SARS-CoV-2 genome

Screening for mutational signatures in the SARS-CoV-2 genome

To investigate the mutational patterns of the SARS-CoV-2 genome we downloaded from GISAID database (https://www.gisaid.org/) 4,672,296 available strains that were distributed globally (*Step 1* in **Figure S7**). These strains were aligned with Bowtie aligner [6], having as reference the Wuhan first assembly NC_045512, obtained from NCBI (https://www.ncbi.nlm.nih.gov/sars-cov-2/) (*Step 2* in **Figure S7**). More information regarding the commands used for the alignments is provided in Part 1 of the supplementary code file (bioinformatic.analysis.sh). The identification of mutations is performed with an "in-house" script (Part 2 of supplementary code file) using calmd function from SAMtools [14], which is based on proteome occupancy profile studies (*Step 3* in **Figure S7**) [15]. Candidate APOBEC sites need to have a C→U frequency of mutation at the same nucleotide position of more than 5 reads. Furthermore, in order to investigate at RNA secondary structure level the filtered deaminated sites (with more than 5 C→U mutation counts), we extracted windows of ±60 nucleotides around the

C→U most frequent sites and interrogated the folding of the RNA sequences using the Vienna RNA fold algorithm (*Step 4* in **Figure S7**) [16]. To guide the RNA folding we incorporated SHAPE reactivities from SHAPE-seq data for SARS-CoV-2 [17]. To decipher the candidate motifs we counted the frequency of letters starting from k-mers of ±5 nucleotides from the most frequent deaminated position up to 30-mers, which is usually taken as an upper limit in most RBP pull-downs and usually corresponds to the protein bound protected fragment [18, 19] (*Step 4* in **Figure S7**). The frequency for each letter was determined via a perl script, which extracts all possible k-mers and their frequencies. The highest motif consensus was around ±5 nucleotides from the C→U deaminated nucleotide, as the motif becomes more degenerate when extending above ±7 nucleotides. Next, the frequency of each k-mer was plotted with Web-logo motifs [20]. In addition position-weight matrices (PWM) for each letter (nucleotide) around the deaminated RNA nucleotides were extracted. A Markov model which extracts the probability of each pairs of nucleotide in each position of the k-mer was also used (i.e. frequency of Adenine in position 1 of the 5mer (A_1) followed by C at position 2, followed by U at position 3), thus a score k-mer matrix is formed demonstrating the probability of each nucleotide per position plus the probability of having specific pairs of di-nucleotides (*Step 4*, right hand panel, in **Figure S7**). The dominant RNA structure motif was identified using the BEAM software [21]. Overall the motif analysis regarding the sequence composition around the most frequently deaminated sites, along with the RNA structure, revealed an UACCA enrichment around regions of open hairpin structures, in agreement with the results from the literature [22-27].

Filtering and alignments of Fastq files

Filtering and alignments of Fastq files for the SARS-CoV2 strains from the infected Vero E6 cells and from actual patients were demultiplexed with Flexbar [3]. Quality control of the Fastq files was assessed with FastQC tools [4]. Adapter sequences were removed with Cutadapt program [5] with the following parameters: quality trimming was set to 28 and the minimum allowed nucleotide length after trimming was 21 nucleotides. Potential, very high over-represented k-mers, at the beginning of the reads, were removed. Alignments were performed with Bowtie2 [6] with the parameter –very-sensitive using as a reference genome the B.1.222 and the B.1 (accession No MT459880.1) SARS-CoV2 strains.

Mutation analysis of other viruses

Data for HIV, HPV, KSHV, and EBV virus strains were downloaded from NCB1 virus (https://www.ncbi.nlm.nih.gov/labs/virus/vssi/#/). Alignments for each virus strain were performed by applying Bowtie 2 [6] and using the following as reference genomes: NC_001802.1 for HIV, NC_009333.1for HPV, NC_009333.1 for KSHV and NC_009334.1 for EBV. Alignments in Bowtie 2 were carried out setting the parameter –very-sensitive. The different strains examined for HIV were 482, 100 for HPV, 12 for KSHV and 15 for EBV. Mutational status analysis for each virus was performed similarly to that followed for APOBEC identification and is explained i**n Step1** of the supplementary code file (bioinformatic.analysis.sh). The identification of mutations was performed with the "in-house" script (**Step 2** of supplementary code file) using in parallel calmd function from SAMtools [14]. The number of mutations observed in the different strains versus the total captured sequenced bases for each of the strains, was plotted for each virus, using

boxplots in R. Statistical significance was determined by applying Wilcoxon rank-sum test with the symbol ** corresponding to statistical significance of p-value<=0.01.

Figure S1: SARS-CoV-2 antibody production and screening selection. A. Workflow of the procedure for antibody production. **B.** Sequel of screening steps for antibody production and selection. **C.** Final screening step processes leading to the selection of G2 monoclonal antibody. Graph depicting the structure of G2 antibody as well as the DNA sequences of FRs and CDRs elements of variable regions.

Suppl Figure 1

A.

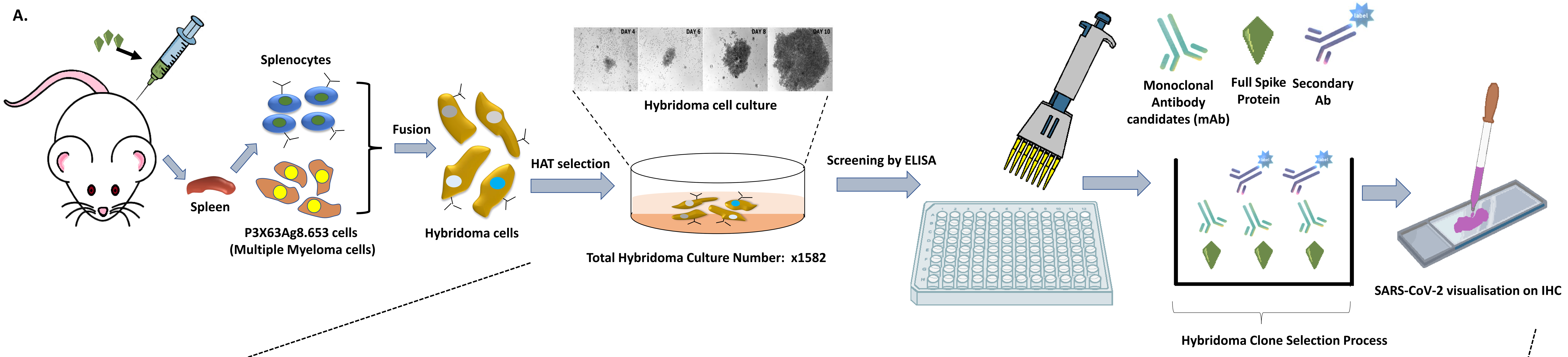

B.

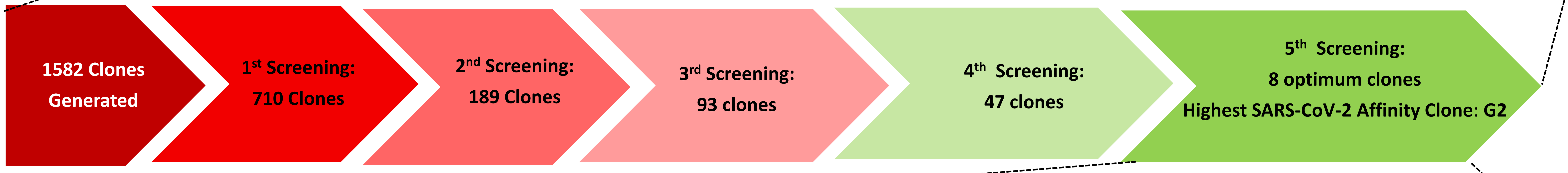

C.

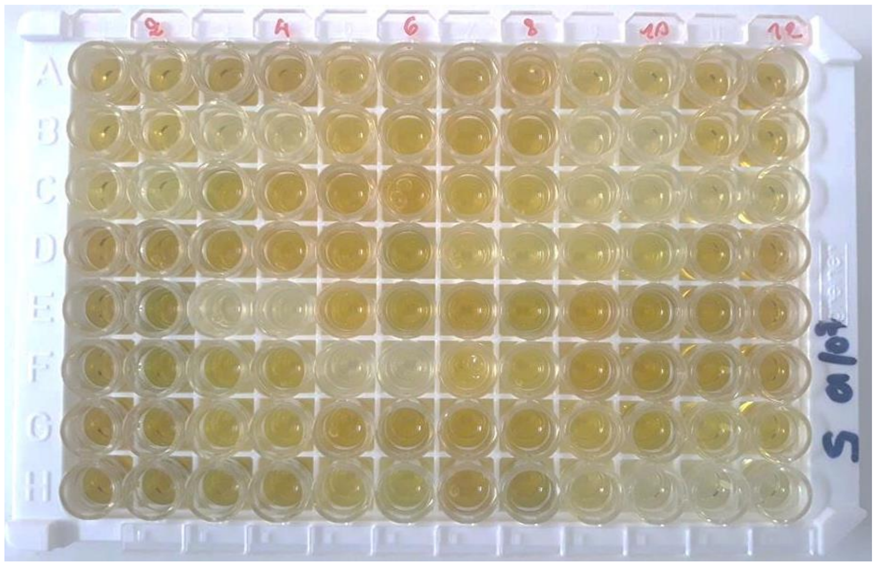

	PBS	RBD	PBS	RBD	PBS	RBD	PBS	RBD	PBS	RBD	PBS	RBD
**A**	G2	G2	H4	H4	D6	D6	D7	D7	D9	D9	A11	A11
**B**	H2	H2	B5	B5	E6	E6	E7	E7	F9	F9	B11	B11
**C**	A4	A4	D5	D5	F6	F6	G7	G7	G9	G9	E11	E11
**D**	B4	B4	F5	F5	G6	G6	H7	H7	A10	A10	F11	F11
**E**	D4	D4	G5	G5	H6	H6	C8	C8	B10	B10	H11	H11
**F**	E4	E4	H5	H5	A7	A7	D8	D8	E10	E10	A12	A12
**G**	F4	F4	A6	A6	B7	B7	E8	E8	F10	F10	G12	G12
**H**	G4	G4	C6	C6	C7	C7	F8	F8	G10	G10	BLANK	BLANK

	1	2	3	4	5	6	7	8	9	10	11	12
**A**	9,999	3,329	9,999	9,999	3,27	9,999	9,999	9,999	1,894	1,816	9,999	9,999
**B**	2,648	2,051	0,485	0,524	2,838	9,999	9,999	9,999	0,476	0,403	2,495	2,753
**C**	1,553	0,94	9,999	9,999	9,999	9,999	3,253	2,818	0,716	0,569	0,539	0,81
**D**	9,999	9,999	9,999	9,999	9,999	9,999	1,366	1,093	1,332	1,728	9,999	9,999
**E**	9,999	9,999	0,362	0,363	9,999	9,999	9,999	9,999	9,999	9,999	9,999	9,999
**F**	9,999	9,999	9,999	9,999	0,384	0,348	2,713	2,172	9,999	9,999	9,999	9,999
**G**	9,999	9,999	2,517	2,657	9,999	9,999	9,999	9,999	2,265	9,999	1,71	2,454
**H**	9,999	9,999	9,999	9,999	2,324	2,419	9,999	9,999	1,026	0,377	0,342	0,398

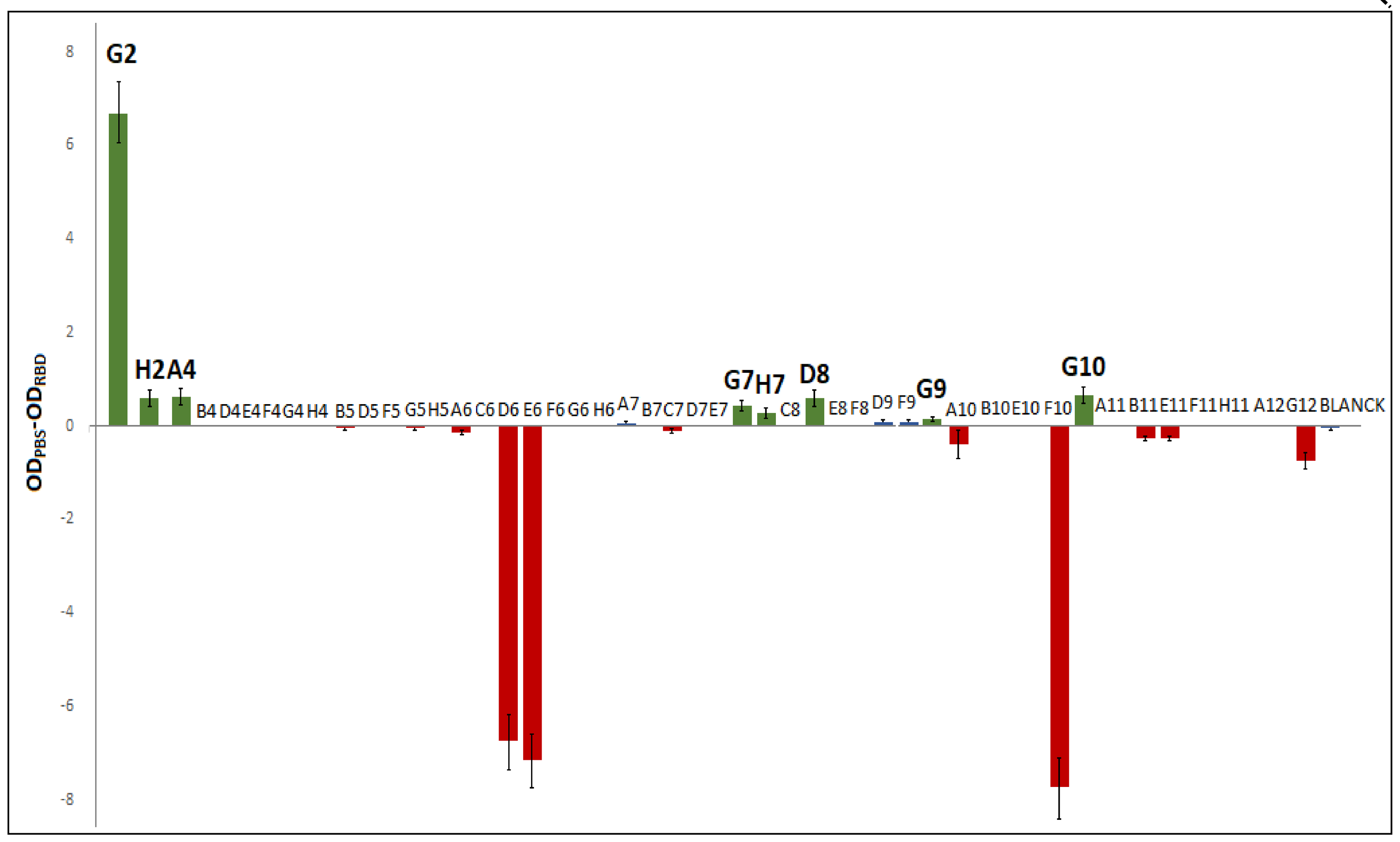

D.

DNA sequence of CDRs and FRs of Light chain
FR1:AACATTATGATGACACAGTCGCCATCATCTCTGGCTGTGTCTGCAGGAGAAAAGGTCACTATGAGCTGTAAGTCCAGTT
CDR1:CAAAGTGTTTTATACAGTTCAAATCAGAAGAACTAC
FR2:TTGGCCTGGTACCAGCAGAAACCAGGGCAGTCTCCTAAACTGCTGATCTAC
CDR2:TGGGCATCC
FR3:ACTAGGGAATCTGGTGTCCCTGATCGCTTCACAGGCAGTGGATCTGGGACAGATTTTACTCTTACCATCAGCAGTGTACAAGCTGAAGACCTGGCAGTTTATAC
CDR3:TGTCATCAATACCTCTCCTCGCTCACGTTC

DNA sequence of CDRs and FRs of Heavy chain
FR1:GAAGTGATGCTGGTGGAGTCTGGGGGAGGCTTAGTGAGGCCTGGAGGGACCCTGAAACTCTCCTGTGCAGCCTCT
CDR1:GGATTCACTTTTAGTGACGCCTGG
FR2:ATGGACTGGGTCCGCCAGTCTCCAGAGAAGGGGCTTGAGTGGGTTGCTGAA
CDR2: ATTAGAAACAAAGCTAATAATCATGCAACA
FR3:TACTATGCTGAGTCTGTGAAAGGGAGGTTCACCATCTCAAGAGATGATTCCAAAAGTAGTGTCTACCTGCAAATGAACAACTTAAGAGCTGAAGACACTGGCATTTATTAC
CDR3:TGTACCAGGCTCTATTACTACGGTAGTACCTACTACTTTGACTACTG
FR4:GGCCAAGGCACCACTCTCACAGTCTCCTCAG

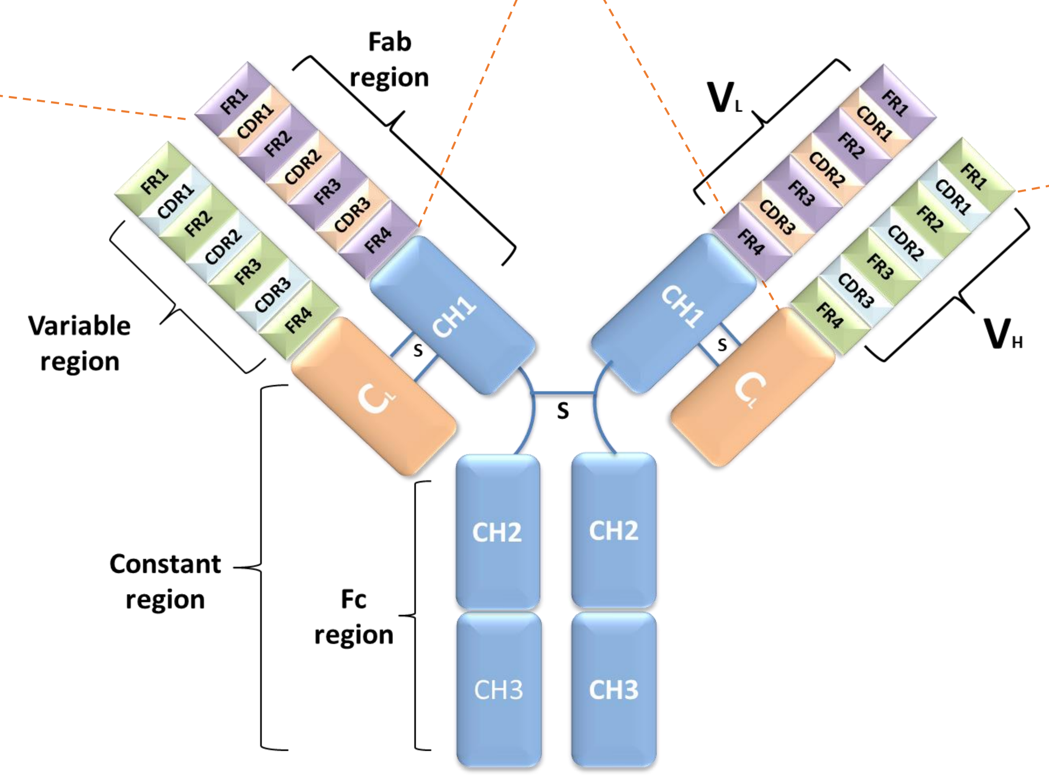

Figure S2: G2 immunostaining in COVID-19 lungs. Representative immunohistochemical results depicting G2 positive and negative staining in small peripheral airways of COVID-19 lungs.

Figure S2

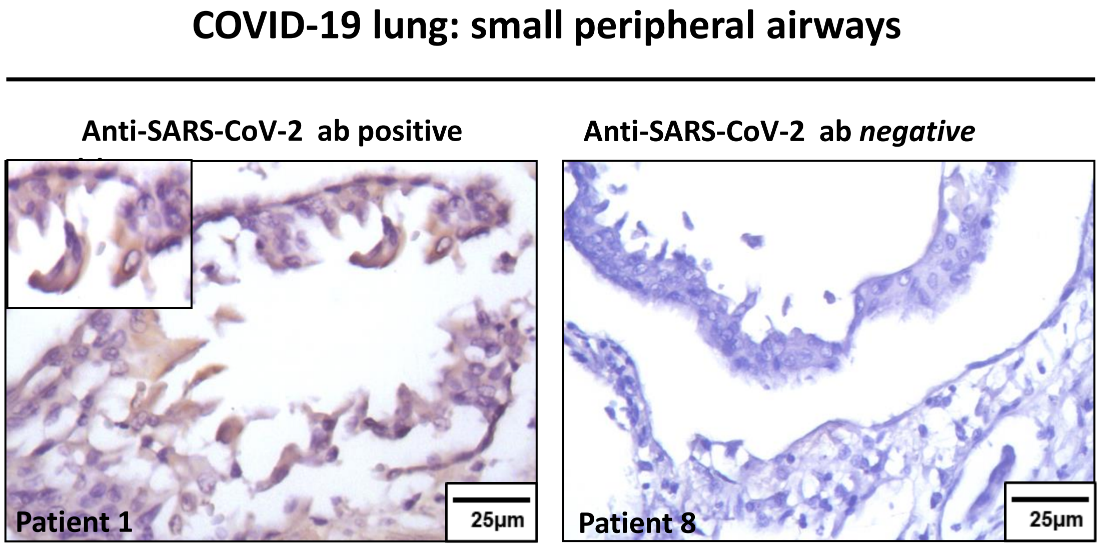
COVID-19 lung: small peripheral airways
Anti-SARS-CoV-2 ab positive
Anti-SARS-CoV-2 ab *negative*
Patient 1
25µm
Patient 8
25µm

Figure S3: Quantification of double anti-SARS-CoV-2 ab (G2)/SenTraGor and anti-SARS-CoV-2 ab (G2)/p16 positive AT-II cells in non COVID19 and COVID19 lung parenchyma. ****p<0.001

Figure S3

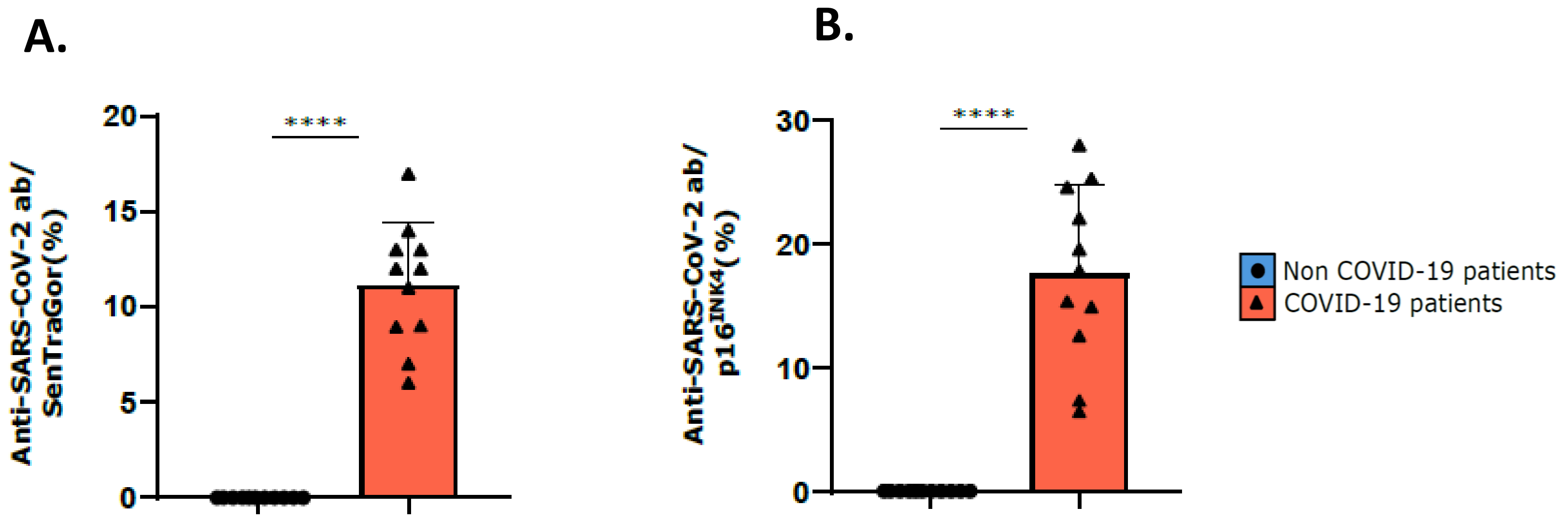
A.
B.
Anti-SARS-CoV-2 ab/ SenTraGor(%)
Anti-SARS-CoV-2 ab/ p16INK4(%)
****
****
20
15
10
5
0
30
20
10
0
Non COVID-19 patients
COVID-19 patients

Figure S4: Immunohistochemcal analysis in acute non COVID-19 pneumonias. Representative images from serial section staining of anti-SARS-CoV-2 ab G2, SenTraGor, ACE-2, TTF-1, IL-6, IL1-β and $p16^{INK4a}$ in the lung parenchyma from acute non COVID-19 pneumonia cases (**A**) and corresponding statistical analysis (**B**). ****p<0.001

Figure S4

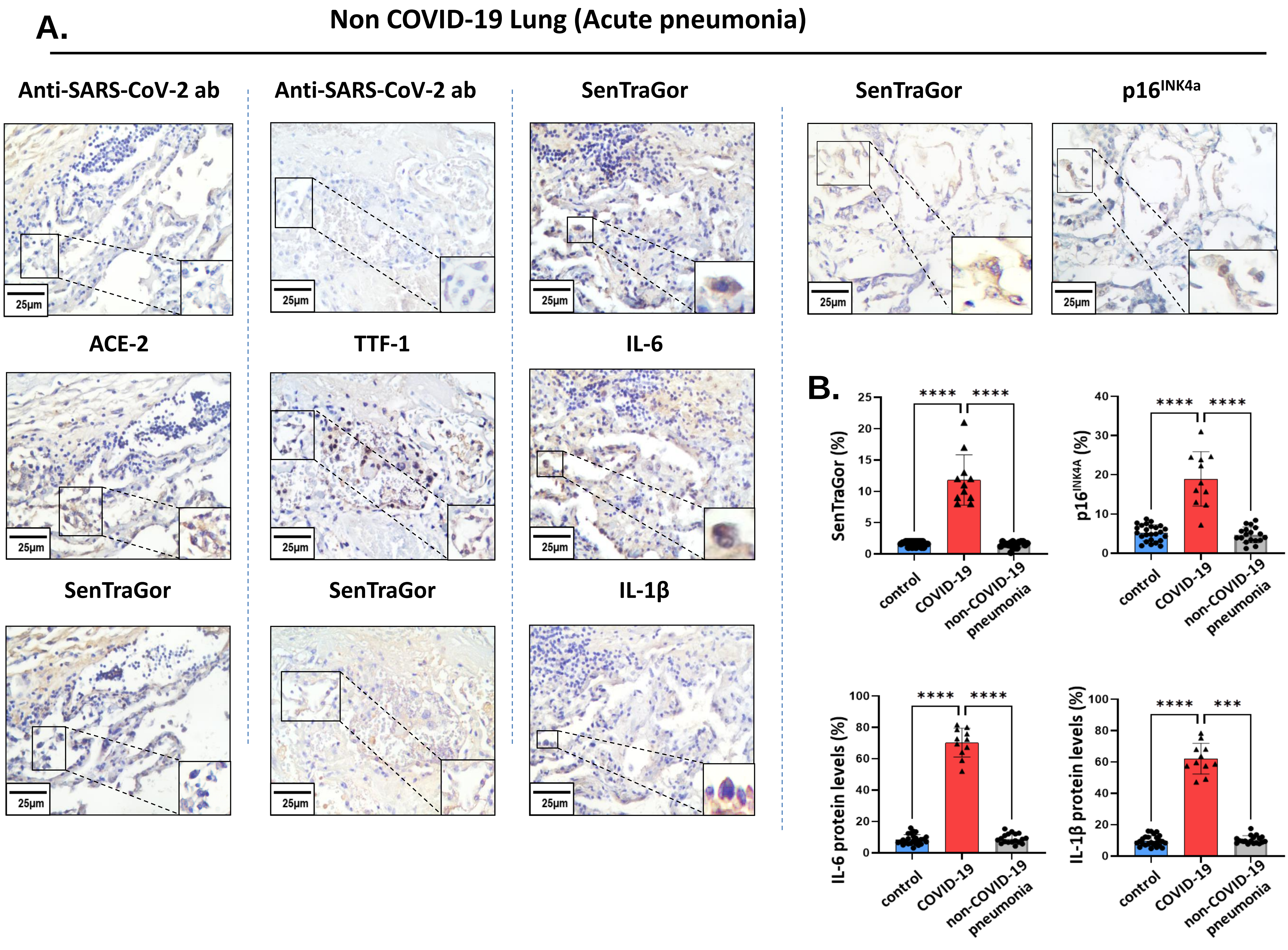

A.
Non COVID-19 Lung (Acute pneumonia)
Anti-SARS-CoV-2 ab
Anti-SARS-CoV-2 ab
SenTraGor
SenTraGor
p16INK4a
ACE-2
TTF-1
IL-6
SenTraGor
SenTraGor
IL-1β
25μm
B.
SenTraGor (%)
p16INK4A (%)
IL-6 protein levels (%)
IL-1β protein levels (%)
control
COVID-19
non-COVID-19 pneumonia
****
***

Figure S5: SARS-CoV-2 induced senescence in Vero cells (A) and in the alveospheres (B). Double immunostaining for SARS-CoV-2 infection/senescence induction (1), senescence induction/cellular proliferation (2) and senescence induction (3). B. Staining for senescence induction (assessed by SenTraGor) in the alveospheres shown in Figure 3. Black arrows depict senescent cells in comparison to non senescent ones (yellow arrow). Absence of senescence is clearly evident in non infected alveolar cells.

Figure S5

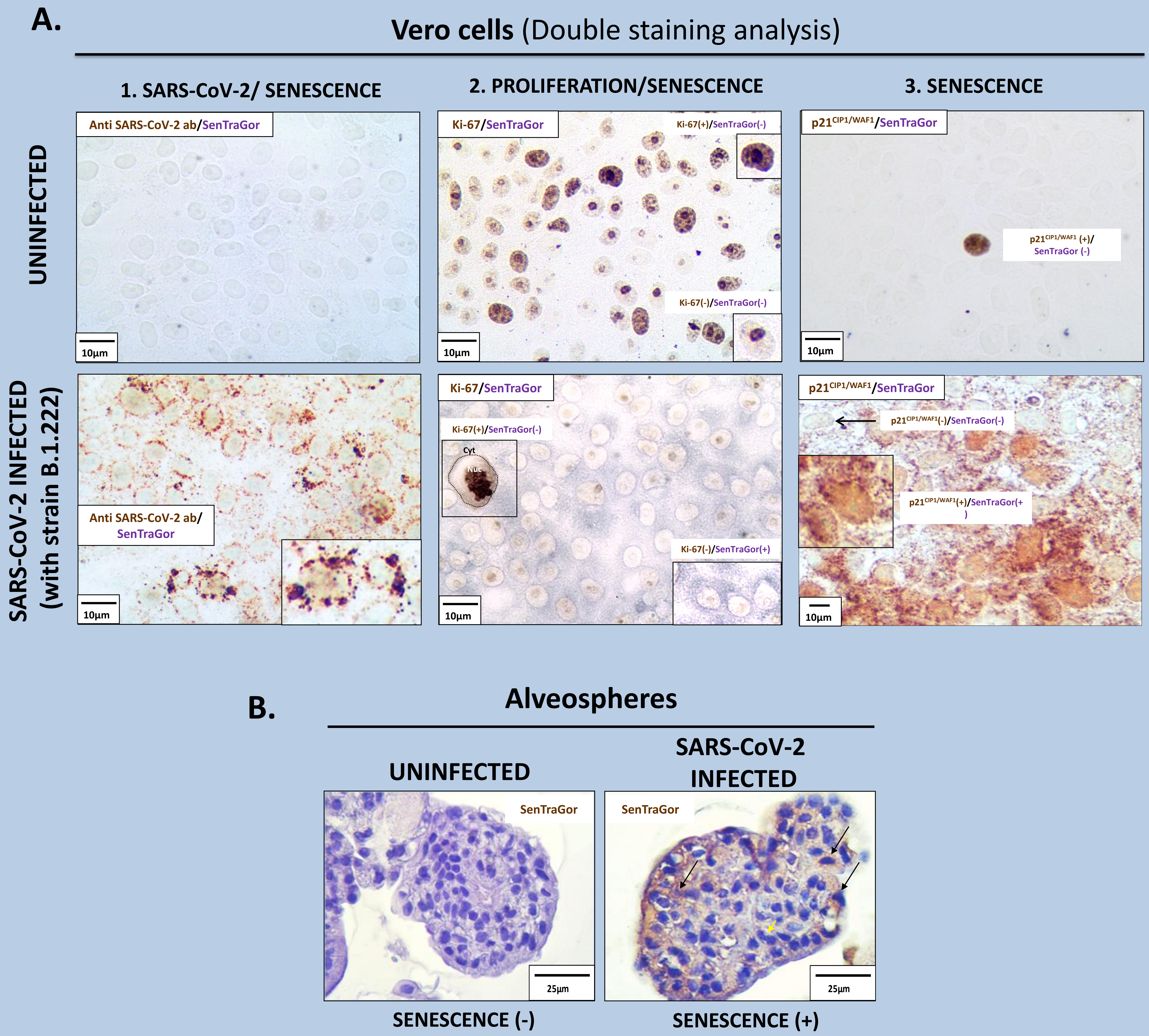
A.
Vero cells (Double staining analysis)
1. SARS-CoV-2/ SENESCENCE
2. PROLIFERATION/SENESCENCE
3. SENESCENCE
UNINFECTED
SARS-CoV-2 INFECTED (with strain B.1.222)
Anti SARS-CoV-2 ab/SenTraGor
Ki-67/SenTraGor
Ki-67(+)/SenTraGor(-)
Ki-67(-)/SenTraGor(-)
Ki-67(-)/SenTraGor(+)
Cyt
Nuc
p21CIP1/WAF1/SenTraGor
p21CIP1/WAF1 (+)/ SenTraGor (-)
p21CIP1/WAF1(-)/SenTraGor(-)
p21CIP1/WAF1(+)/SenTraGor(+)
10μm
B.
Alveospheres
UNINFECTED
SARS-CoV-2 INFECTED
SenTraGor
25μm
SENESCENCE (-)
SENESCENCE (+)

Figure S6: ATM inhibition in Infected Vero E6 cells results in senescence suppression. Treated, with the ATM inhibitor KU-55933, Vero cells exhibit dramatic decrease in senescence assessed by SenTraGor staining (graph, $p<0.0001$).

Figure S6

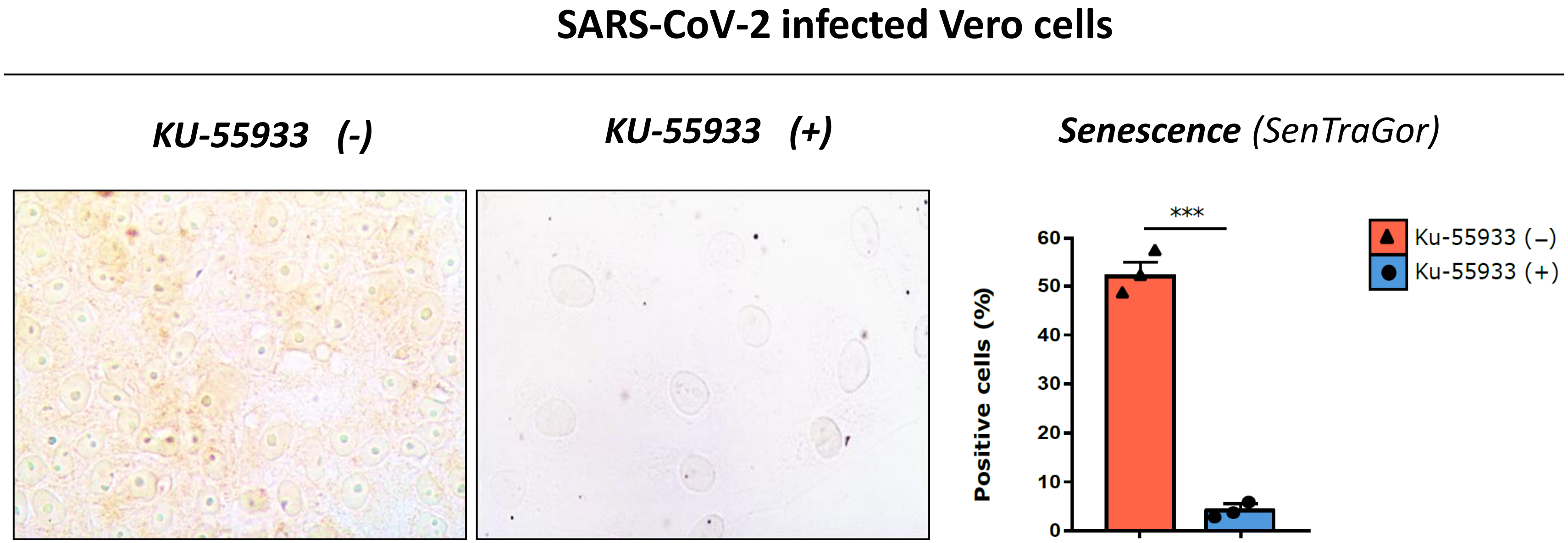
SARS-CoV-2 infected Vero cells
KU-55933 (-)
KU-55933 (+)
Senescence (SenTraGor)
***
Positive cells (%)
0
10
20
30
40
50
60
Ku-55933 (–)
Ku-55933 (+)

Figure S7: Flow diagram of bioinformatic steps followed for the identification of APOBEC mutational signatures in the SARS-CoV-2 genome. From GISAID database (https://www.gisaid.org/) 4,672,296 available strains were downloaded (Step 1), aligned against the Wuhan first assembly NC_045512 (https://www.ncbi.nlm.nih.gov/sars-cov-2/) as reference (Step 2), and mutations were identified followed by recall of C→U mutation with >5 NGS read depths (Step 3). Flanking sequences (*k-mers*) around the recorded C→U mutations were interrogated at linear and secondary conformation (2D) level (see online supplemental Materials and Methods for details) and verified against experimentally validated APOBEC motifs to confirm C→U substitutions that are APOBEC-mediated. NGS: Next Generation Sequencing.

Figure S7

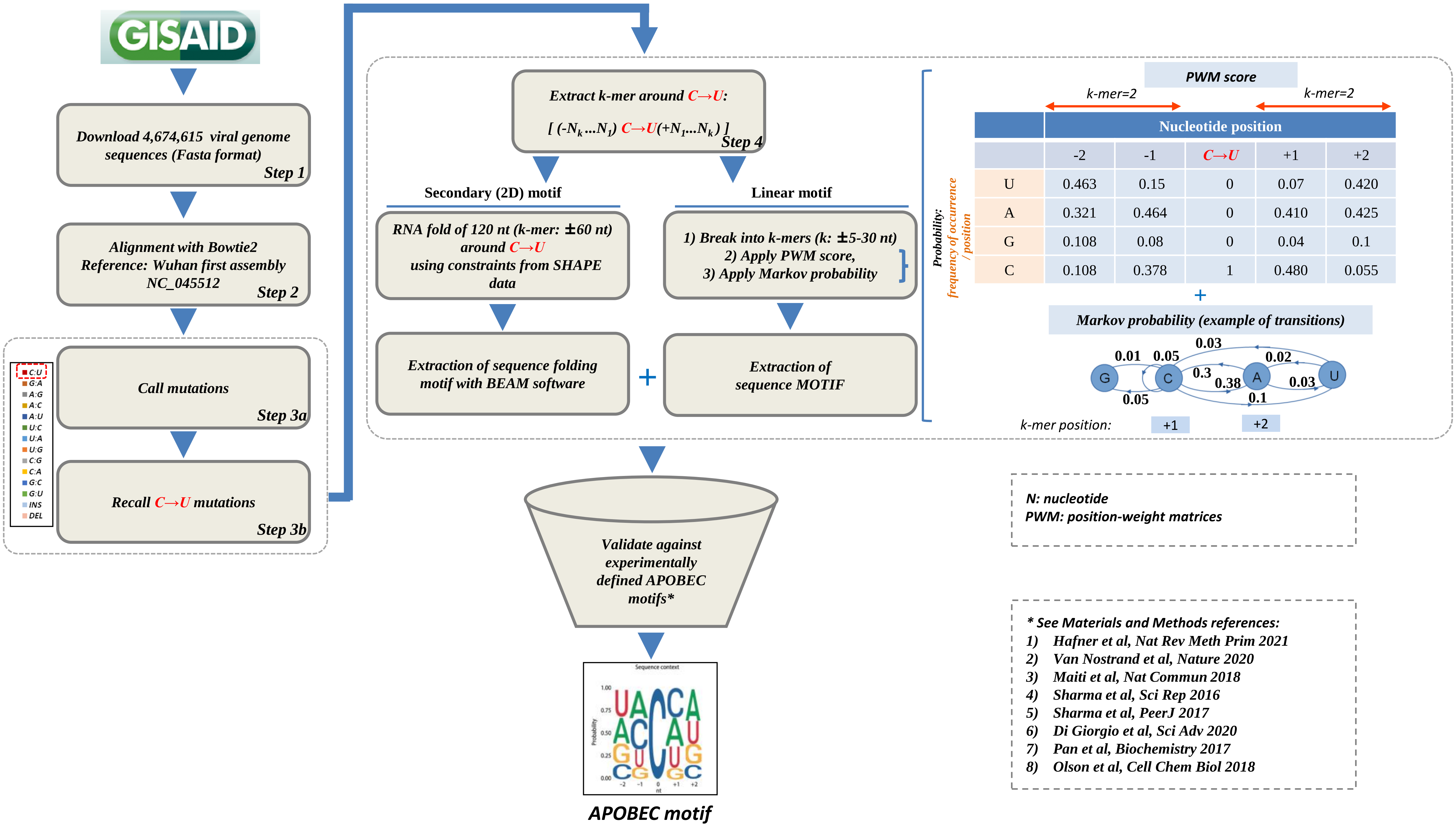

	Nucleotide position				
	-2	-1	C→U	+1	+2
U	0.463	0.15	0	0.07	0.420
A	0.321	0.464	0	0.410	0.425
G	0.108	0.08	0	0.04	0.1
C	0.108	0.378	1	0.480	0.055

Figure S8: Hypothetical model: Features and mechanistic insights of SARS-CoV-2 induced senescence. Briefly, SARS-CoV-2 entrance results in activation of stress response signaling pathways. The virus hijacks certain cellular functions linked with RNA processing, translation and endoplasmic reticulum turnover causing increased energy consumption and eventually an energy shift. Increased ROS production and oxidative stress lead to DNA damage and DNA damage response activation. In addition, DDR and the subsequent cell cycle arrest is also driven by an interaction of Coronavirus nsp13 protein and DNA polymerase δ and SASP via the cGAS/STING and other DNA damage dependent pathways.

Figure S8

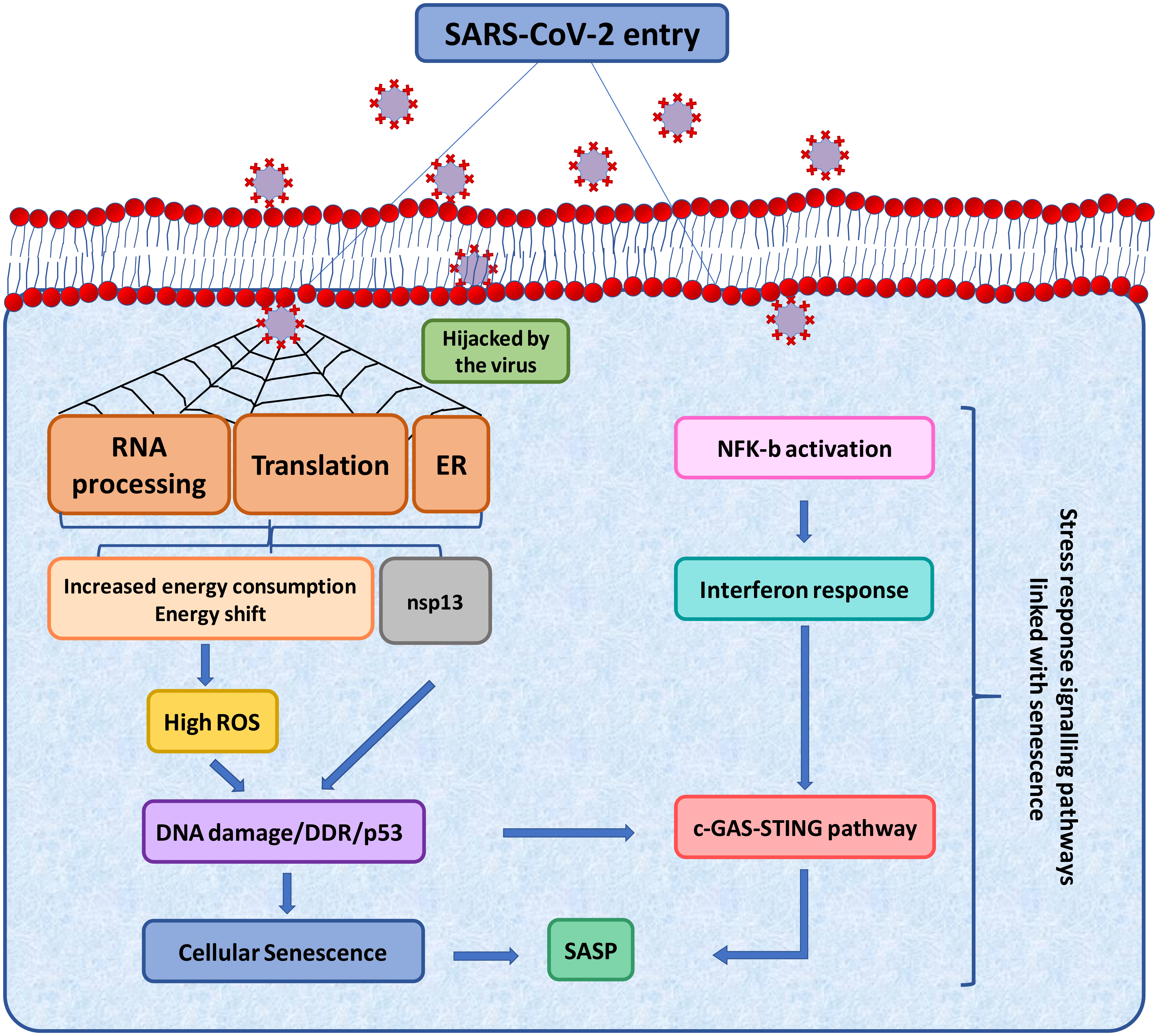

Abbreviations. SARS-CoV-2: Severe acute respiratory syndrome coronavirus 2; ER: endoplasmic reticulum; ROS: Reactive Oxygen Species; DDR: DNA Damage Response; SASP: senescence-associated secretory phenotype.

References
- Hekman et al, Mol Cell 2020
- Lee et al, Nature 2021
- Victor et al, Biochem Biophys Res Com 2021
- Bartkova et al, Nature 2006
- Gorgoulis et al, Cell 2019

Figure S9: APOBEC-mediated mutations in the genome of the omicron (B.1.1.529) SARS-CoV-2 variant of concern. Histogram depicts the predominance of C→U substitutions in the genome of omicron strains deposited in the GISAID database. The majority of these mutations are APOBEC derived (pie chart), following application of the algorithm of Figure S7.

Figure S9

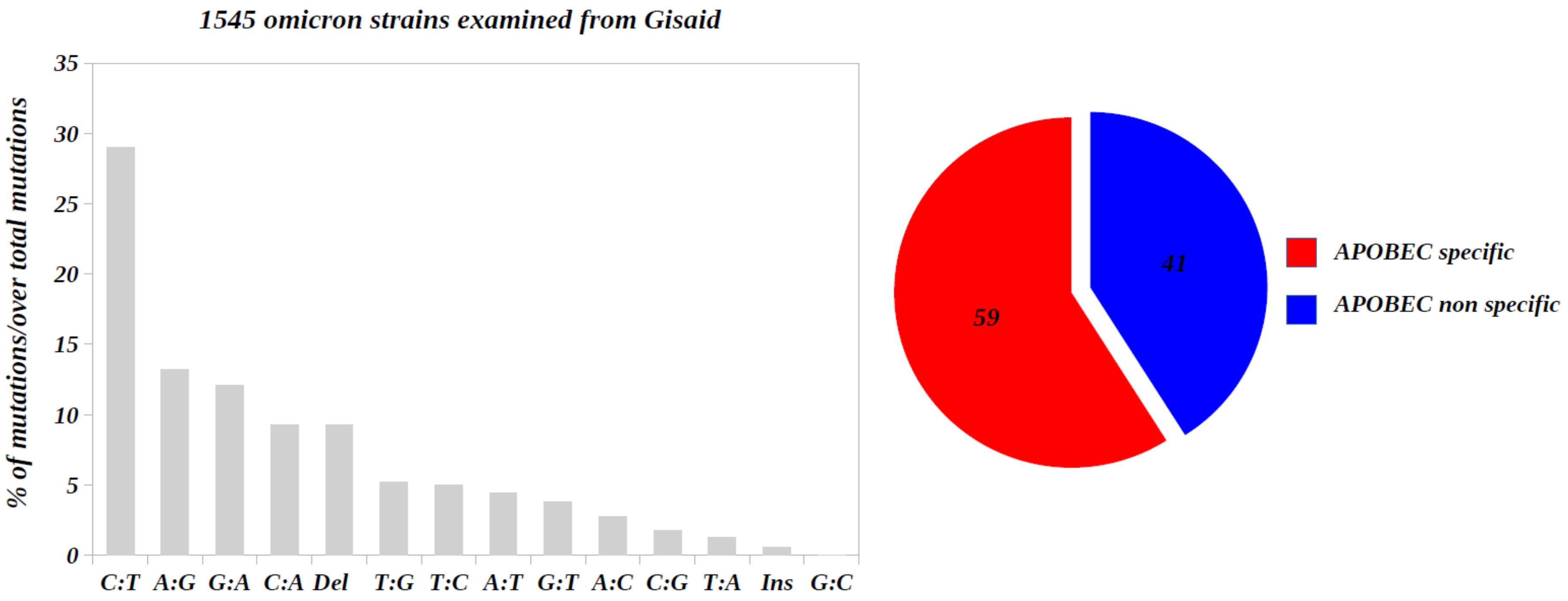

1545 omicron strains examined from Gisaid
% of mutations/over total mutations
35
30
25
20
15
10
5
0
C:T
A:G
G:A
C:A
Del
T:G
T:C
A:T
G:T
A:C
C:G
T:A
Ins
G:C
59
41
APOBEC specific
APOBEC non specific

Figure S10: Differences in mutation rates between viruses inducing and bypassing senescence. Box plots depicting that viruses bypassing senescence exhibit significantly lower mutation rates in comparison to viruses inducing senescence. **p<0.01

Figure S10

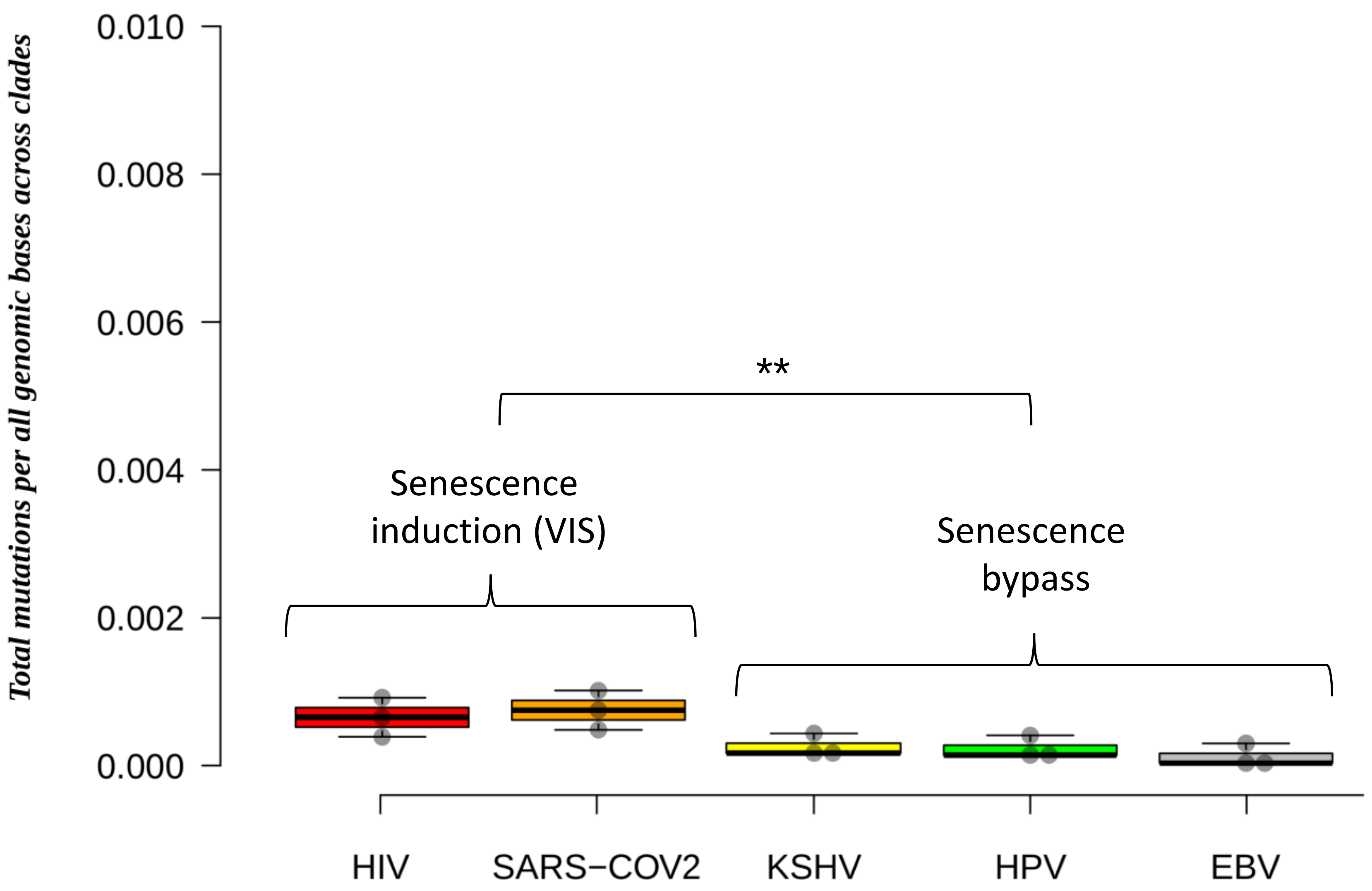
Total mutations per all genomic bases across clades
0.010
0.008
0.006
0.004
0.002
0.000
**
Senescence induction (VIS)
Senescence bypass
HIV
SARS−COV2
KSHV
HPV
EBV

Suppl Table S1: Clinicopathological characteristics of COVID-19 and non-COVID control cases examined for G2 antibody, senescence, IL-6 and IL-1b, and evaluation results in the corresponding lung tissues.

SUPPL TABLE S1: CLINICOPATHOLOGICAL CHARACTERISTICS OF COVID AND NON COVID-19 CASES

Suppl Table S1a: Clinicopathological characteristics of COVID-19 cases examined along with G2 antibody, anti-S antibody (GENETEK), senescence, IL-6 and IL-1β evaluation results in the corresponding lung tissues

CASE	SEX	AGE	DATE OF DEATH	DATE OF COVID-19 TEST	HOSPITALIZED DAYS	DAYS IN INTENSIVE CARE UNIT	RESPIRATORY ASSISTANCE	TREATMENTS	COMORBIDITIES	MOLECULAR TEST	G2 EVALUATION		COMERCIAL ANTI-S AB (GENETEK) EVALUATION		SenTraGor EVALUATION (%)	P16 (%)	IL-6 (%)	IL-1b (%)
											Schaefer et al, 20120	Remmelink et al, 2020	Schaefer et al, 20120	Remmelink et al, 2020				
A2000026	F	73	05/03/2020	01/03/2020	4	-	NO	Kaletra, Remdesevir	Follicular lymphoma grade 1-2. Mixed type cardiopathy on chemotherapy	Positive	<5/4 mm² (+)	++	<5/4 mm² (+)	++	9	12.3	80	57.42
A2000036	M	87	20/03/2020	16/03/2020	5	4	YES	Reyataz,Co-amoxiciline	Type 2 non-insulin dependent diabetes. Dyslipidemia	Positive	>5-50/4 mm² (+++)	+++	>5-50/4 mm² (+++)	+++	17	24.47	52	59.65
A2000038	F	74	21/03/2020	12/03/2020	9	5	YES	Pipéracilline + tazobactam Atanazavir + hydroxychloroquine Remdesevir	COPD stage Iib. Pacemacer for rhythmic cardiopathy. Non-smoker	Positive	5-50/4 mm² (++)	+++	5-50/4 mm² (++)	+++	12	15.71	72.72	68.12
A2000042	M	80	28/03/2020	16/03/2020	13	13	YES	Augmentin+ventolin Tazobactam Plaquénil + Reyataz	COPD stage II	Positive	5-50/4 mm² (++)	+++	5-50/4 mm² (++)	+++	13	22.32	69.47	75.32
A2000045	M	96	01/04/2020	24/03/2020	8	8	NO	Co-amoxicilline Procalcitonine Hydroxychloroquine	Chronic renal failure. Pulmonary embolism (2019).	Positive	<5/4 mm² (+)	++	<5/4 mm² (+)	++	10	17.97	81.62	68
A2000047	M	75	02/04/2020	10/03/2020	7	7	YES		Hypertrophic cardiomyopathy. Pacemaker for complete atrioventricular block. Type 2 non-insulin dependent diabetes. Overweight.	Positive	5-50/4 mm² (++)	+++	5-50/4 mm² (++)	+++	11	24.66	67.3	47.22
A2000048	M	36	02/04/2020	01/04/2020	2	2	YES		Tetraplegia. Post-anoxic encephalopathy. Aspiration pneumonia	Weakly positive	<5/4 mm² (+)	++	<5/4 mm² (+)	++	9	16.04	64.14	49.1
A2000052	M	61	16/04/2020	01/04/2020	18	18	YES	Vancomycine + rifampicine Imipénème Noradrénaline	Ischemic heart disease. Hypertension. Type 2 non-insulin dependent diabetes. Peripheral artery disease (PAD). COPD with asthmatic component. Alcohol dependent syndrome. Possible hemochromatosis.	Weakly positive	<5/4 mm² (+)	++	<5/4 mm² (+)	++	8	23.87	78.55	63.67
A2000058	F	72	27/04/2020	26/04/2020	4	4	NO	Co-amoxicilline Pipéracilline – Tazobactam Furosémide	Hypertension. Chronic renal failure. COPD, former smoker. Bipolar disorder under long-term lithium treatment. Peripheral artery disease (PAD)	Positive	5-50/4 mm² (++)	+++	5-50/4 mm² (++)	+++	12	13	72.36	78.4
A2000062	M	70	07/05/2020	n.c.	34	26	YES	Co-amoxicilline Hydrochloroquine Céfépime Dobutamine Pipéracilline-Tazobactam Méropénème Lévosimendan Aspirine et Clopidogrel Levetiracetam Pipéracilline-Tazobactam et Vancomicine	Ischemic and valvular heart disease. Hypertension.	Weakly positive	<5/4 mm² (+)	++	<5/4 mm² (+)	++	8	7.2	58.96	57.43
3403/20 B2	M	56	11/12/2020	02/12/2020	10	6	YES	Dexamethasone, Pregabalin, Insulin therapy, Piperacillin-Tazobactam, Vancomycin, Azithromycin	Decompensated Liver Cirrhosis, Hemophilia A, Type 2 Diabetes, Brain mass under investigation	Positive	>5-50/4 mm² (+++)	+++	>5-50/4 mm² (+++)	+++	21	31	75.9	58.72

SUPPL TABLE S1: CLINICOPATHOLOGICAL CHARACTERISTICS OF COVID AND NON COVID-19 CASES

Suppl Table S1b: Clinicopathological characteristics of non COVID-19 cases examined along with senescence, IL-6 and IL-1β evaluation results in the corresponding lung tissues

CASE	SEX	AGE	SMOKING	HISTOLOGY	GRADE	T	N	M	STAGE	COMORBIDITIES	SenTraGor EVALUATION (%)	P16 (%)	IL-6 (%)	IL-1b (%)
K14	F	72	M	1	3	1	0	0	IA		1	3.2	8.52	9.36
K22	M	84	H	1	2	2	0	0	IB	COPD	2	4.7	13.47	15.78
D30	F	76	H	1	3	2	0	0	IB	COPD	2	5.2	5.78	7.36
D8	M	80	H	1	3	3	2	0	IIIA		2	7.1	7	5.14
D19	M	84	M	2	3	2	0	0	IB	COPD	1.6	1.93	15.87	14.36
D23	M	76	M	1	2	1	1	0	IIA	Hypertension, Diabetes	1	8.74	10.63	12
A23	M	42	M	1	3	2	2	0	IIIA		2	4.31	9.4	11.68
D52	M	61	H	2	3	2	0	0	IIA	Coronary artery disease, COPD	1.8	6.14	12.36	15.92
K53	F	72	H	5	2	1	0	0	IA		1	4.92	6.87	9.87
K50	M	70	M	2	2	1	1	0	NA	Hypertension	1.8	7.55	4.82	7.84
K42	M	57	H	2	3	2	0	0	IB	COPD	2	6	8.7	8.7
K38	F	44	M	1	3	2	0	0	IB	Hypertension	1.6	7.7	7.6	8.5
B12	M	66	H	2	3	2	0	0	IB	Coronary artery disease, COPD	2	4.3	3.1	5.6
K41	M	69	H	1	2	2	1	0	IIB	Hypertension, Diabetes	1.8	2.6	12.54	11
D27	M	43	M	2	2	2	0	0	IB		1	6.8	5.9	8.4
D13	M	77	H	5	4	2	0	0	IB	COPD	2	2.2	6	4.8
D36	M	80	H	2	2	2	0	0	IB	Coronary artery disease, COPD	2	5.4	13.8	9.36
K26	F	79	H	1	2	1	0	0	IA	COPD	1.6	1.8	6.5	5.5
D35	M	73	H	1	3	2	0	0	IB		1.6	6.4	5	7.7
K19	F	69	M	2	3	2	1	0	IIB	COPD	1.6	7	7.8	12.2
D28	M	76	H	2	2	2	1	0	IIB	COPD	1.6	8.1	10.14	8.4
D20	M	75	H	1	3	1	1	0	IIA	Coronary artery disease, COPD	1.8	3.4	8.3	7
D44	F	63	M	2	3	2	0	0	IB		1	2.9	5.5	6.6
B10	M	77	H	1	2	2	1	0	IIB	COPD	2	4.8	9	15.52
K19	F	78	M	2	3	2	1	0	IIB	Coronary artery disease, COPD	2	6.5	7	13
AP1	M	70	H	6						Stroke, Hypertension, Diabetes	1	4.95	8.7	9.36
AP2	M	77	H	6						Stroke, Hypertension, Diabetes	2	6	9.4	12.2
AP3	M	84	H	6						Stroke, Cardiac and renal failure	1.6	7.6	12	11.4
AP4	F	81	H	6						Stroke, Coronary artery disease	2	8.4	13.5	11.92
AP5	M	71	H	6						Stroke, Diabetes	1.2	5.6	7.80	9.60
AP6	M	69	H	6						Stroke	0.9	7.43	12.50	9.50
AP7	F	74	H	6						Stroke, Diabetes	0.2	3.4	4.30	8.00
AP8	F	79	M	6						Stroke, Coronary artery disease	2	6.5	6.90	11.20
AP9	M	83	M	6						Stroke, Cardiac failure, Anemia	1.4	1.2	8.20	13.40
AP10	M	84	H	6						Stroke, Cardiac failure	1.6	3.9	5.70	8.40
AP11	M	72	M	6						Stroke, Coronary artery disease, Lupus	1.9	4	11.00	9.90
AP12	F	77	H	6						Stroke, Coronary artery disease	1.8	5.3	15.30	17.50
AP13	F	63	H	6						Stroke, Coronary artery disease, Diabetes	1.5	3.3	6.70	7.60
AP14	M	83	M	6						Stroke, Hypertension, Diabetes	0.9	1.7	7.10	8.20
AP15	F	76	M	6						Stroke, Coronary artery disease, Diabetes	1.4	2.8	9.20	9.60
AP16	F	67	H	6						Stroke, Coronary artery disease	1.7	3.9	6.70	8.80
AP17	F	79	H	6						Stroke, Diabetes, Cardiac failure	1.6	4.8	5.90	7.90
AP18	M	70	M	6						Stroke, Coronary artery disease	2	3.2	12.00	13.60

SUPPL TABLE S1: CLINICOPATHOLOGICAL CHARACTERISTICS OF COVID AND NON COVID-19 CASES

**Smoking**	Medium: 30-50 cigarretes/day	**Histology**	1. Squamous carcinoma
	Heavy: > 50 cigarettes/day		2. Adenocarcinoma
			3. Large Cell carcinoma
			4. Undifferentiated carcinoma
			5. Other carcinoma type
			6. Acute (aspiration) pneumonia
			NA. non-available

References

1. Huan CC, Wang HX, Sheng XX, Wang R, Wang X, Liao Y, Liu QF, Tong GZ, Ding C, Fan HJ, Wu JQ, Mao X. Porcine epidemic diarrhea virus nucleoprotein contributes to HMGB1 transcription and release by interacting with C/EBP-β. *Oncotarget* 2016: 7(46): 75064-75080.
2. Komseli ES, Pateras IS, Krejsgaard T, Stawiski K, Rizou SV, Polyzos A, Roumelioti FM, Chiourea M, Mourkioti I, Paparouna E, Zampetidis CP, Gumeni S, Trougakos IP, Pefani DE, O'Neill E, Gagos S, Eliopoulos AG, Fendler W, Chowdhury D, Bartek J, Gorgoulis VG. A prototypical non-malignant epithelial model to study genome dynamics and concurrently monitor micro-RNAs and proteins in situ during oncogene-induced senescence. *BMC genomics* 2018: 19(1): 37.
3. Dodt M, Roehr JT, Ahmed R, Dieterich C. FLEXBAR-Flexible Barcode and Adapter Processing for Next-Generation Sequencing Platforms. *Biology* 2012: 1(3): 895-905.
4. Andrews S. FastQC: a quality control tool for high throughput sequence data. http://www.bioinformatics.babraham.ac.uk/projects/fastqc, 2010.
5. Martin M. Cutadapt removes adapter sequences from high-throughput sequencing reads. *2011* 2011: 17(1): 3.
6. Langmead B, Salzberg SL. Fast gapped-read alignment with Bowtie 2. *Nat Methods* 2012: 9(4): 357-359.
7. Bolotin DA, Poslavsky S, Davydov AN, Frenkel FE, Fanchi L, Zolotareva OI, Hemmers S, Putintseva EV, Obraztsova AS, Shugay M, Ataullakhanov RI, Rudensky AY, Schumacher TN, Chudakov DM. Antigen receptor repertoire profiling from RNA-seq data. *Nature biotechnology* 2017: 35(10): 908-911.
8. Yang J, Yan R, Roy A, Xu D, Poisson J, Zhang Y. The I-TASSER Suite: protein structure and function prediction. *Nat Methods* 2015: 12(1): 7-8.
9. Evangelou K, Lougiakis N, Rizou SV, Kotsinas A, Kletsas D, Muñoz-Espín D, Kastrinakis NG, Pouli N, Marakos P, Townsend P, Serrano M, Bartek J, Gorgoulis VG. Robust, universal biomarker assay to detect senescent cells in biological specimens. *Aging cell* 2017: 16(1): 192-197.
10. Gorgoulis VG, Vassiliou LV, Karakaidos P, Zacharatos P, Kotsinas A, Liloglou T, Venere M, Ditullio RA, Jr., Kastrinakis NG, Levy B, Kletsas D, Yoneta A, Herlyn M, Kittas C, Halazonetis TD. Activation of the DNA damage checkpoint and genomic instability in human precancerous lesions. *Nature* 2005: 434(7035): 907-913.
11. Schaefer IM, Padera RF, Solomon IH, Kanjilal S, Hammer MM, Hornick JL, Sholl LM. In situ detection of SARS-CoV-2 in lungs and airways of patients with COVID-19. *Modern pathology : an official journal of the United States and Canadian Academy of Pathology, Inc* 2020: 33(11): 2104-2114.
12. Remmelink M, De Mendonça R, D'Haene N, De Clercq S, Verocq C, Lebrun L, Lavis P, Racu ML, Trépant AL, Maris C, Rorive S, Goffard JC, De Witte O, Peluso L, Vincent JL, Decaestecker C, Taccone FS, Salmon I. Unspecific post-mortem findings despite multiorgan viral spread in COVID-19 patients. *Critical care (London, England)* 2020: 24(1): 495.
13. van Vliet T, Varela-Eirin M, Wang B, Borghesan M, Brandenburg SM, Franzin R, Evangelou K, Seelen M, Gorgoulis V, Demaria M. Physiological hypoxia restrains the senescence-associated secretory phenotype via AMPK-mediated mTOR suppression. *Molecular cell* 2021: 81(9): 2041-2052.e2046.
14. Li H, Handsaker B, Wysoker A, Fennell T, Ruan J, Homer N, Marth G, Abecasis G, Durbin R. The Sequence Alignment/Map format and SAMtools. *Bioinformatics* 2009: 25(16): 2078-2079.
15. Schueler M, Munschauer M, Gregersen LH, Finzel A, Loewer A, Chen W, Landthaler M, Dieterich C. Differential protein occupancy profiling of the mRNA transcriptome. *Genome Biol* 2014: 15(1): R15.
16. Gruber AR, Lorenz R, Bernhart SH, Neuböck R, Hofacker IL. The Vienna RNA websuite. *Nucleic Acids Res* 2008: 36(Web Server issue): W70-74.

17. Manfredonia I, Nithin C, Ponce-Salvatierra A, Ghosh P, Wirecki TK, Marinus T, Ogando NS, Snijder EJ, van Hemert MJ, Bujnicki JM, Incarnato D. Genome-wide mapping of SARS-CoV-2 RNA structures identifies therapeutically-relevant elements. *Nucleic Acids Res* 2020: 48(22): 12436-12452.
18. Hafner M, Katsantoni M, Köster T, Marks J, Mukherjee J, Staiger D, Ule J, Zavolan M. CLIP and complementary methods. *Nature Reviews Methods Primers* 2021: 1(1): 20.
19. Van Nostrand EL, Freese P, Pratt GA, Wang X, Wei X, Xiao R, Blue SM, Chen JY, Cody NAL, Dominguez D, Olson S, Sundararaman B, Zhan L, Bazile C, Bouvrette LPB, Bergalet J, Duff MO, Garcia KE, Gelboin-Burkhart C, Hochman M, Lambert NJ, Li H, McGurk MP, Nguyen TB, Palden T, Rabano I, Sathe S, Stanton R, Su A, Wang R, Yee BA, Zhou B, Louie AL, Aigner S, Fu XD, Lécuyer E, Burge CB, Graveley BR, Yeo GW. A large-scale binding and functional map of human RNA-binding proteins. *Nature* 2020: 583(7818): 711-719.
20. Crooks GE, Hon G, Chandonia JM, Brenner SE. WebLogo: a sequence logo generator. *Genome Res* 2004: 14(6): 1188-1190.
21. Pietrosanto M, Mattei E, Helmer-Citterich M, Ferrè F. A novel method for the identification of conserved structural patterns in RNA: From small scale to high-throughput applications. *Nucleic Acids Res* 2016: 44(18): 8600-8609.
22. Maiti A, Myint W, Kanai T, Delviks-Frankenberry K, Sierra Rodriguez C, Pathak VK, Schiffer CA, Matsuo H. Crystal structure of the catalytic domain of HIV-1 restriction factor APOBEC3G in complex with ssDNA. *Nat Commun* 2018: 9(1): 2460.
23. Sharma S, Patnaik SK, Taggart RT, Baysal BE. The double-domain cytidine deaminase APOBEC3G is a cellular site-specific RNA editing enzyme. *Scientific reports* 2016: 6: 39100.
24. Sharma S, Baysal BE. Stem-loop structure preference for site-specific RNA editing by APOBEC3A and APOBEC3G. *PeerJ* 2017: 5: e4136.
25. Di Giorgio S, Martignano F, Torcia MG, Mattiuz G, Conticello SG. Evidence for host-dependent RNA editing in the transcriptome of SARS-CoV-2. *Sci Adv* 2020: 6(25): eabb5813.
26. Pan Y, Sun Z, Maiti A, Kanai T, Matsuo H, Li M, Harris RS, Shlyakhtenko LS, Lyubchenko YL. Nanoscale Characterization of Interaction of APOBEC3G with RNA. *Biochemistry* 2017: 56(10): 1473-1481.
27. Olson ME, Harris RS, Harki DA. APOBEC Enzymes as Targets for Virus and Cancer Therapy. *Cell Chem Biol* 2018: 25(1): 36-49.
