# Supplementary material for: Pulmonary infection by SARS-CoV-2 induces senescence accompanied by an inflammatory phenotype in severe COVID-19: possible implications for viral mutagenesis
Source: Eur Respir J. 2022 Aug 18;60(2):2102951. doi: 10.1183/13993003.02951-2021 (PMC8796696; doi:10.1183/13993003.02951-2021)
Supplement: Supplementary file 2 [file ERJ-02951-2021.Shareable.pdf]

SHAREABLE PDF

Pulmonary infection by SARS-CoV-2 induces senescence accompanied by an inflammatory phenotype in severe COVID-19: possible implications for viral mutagenesis

Konstantinos Evangelou[1,17], Dimitris Veroutis[1,2,17], Koralia Paschalaki[3,17], Periklis G. Foukas[4], Nefeli Lagopati[1,2], Marios Dimitriou[4,5], Angelos Papaspyropoulos[1,2], Bindu Konda[6], Orsalia Hazapis[1], Aikaterini Polyzou[1], Sophia Havaki[1], Athanassios Kotsinas[1], Christos Kittas[1,7], Athanasios G. Tzioufas[8], Laurence de Leval[9], Demetris Vassilakos[1], Sotirios Tsiodras[10,11], Barry R. Stripp[6], Argyris Papantonis[12,13], Giovanni Blandino[14], Ioannis Karakasiliotis[5], Peter J. Barnes[3,18] and Vassilis G. Gorgoulis[1,2,7,15,16,18]

[1]Molecular Carcinogenesis Group, Dept of Histology and Embryology, Medical School, National and Kapodistrian University of Athens, Athens, Greece. [2]Biomedical Research Foundation, Academy of Athens, Athens, Greece. [3]National Heart and Lung Institute, Imperial College London, London, UK. [4]2nd Dept of Pathology, Attikon University Hospital, Medical School, National and Kapodistrian University of Athens, Athens, Greece. [5]Laboratory of Biology, Dept of Medicine, Democritus University of Thrace, Alexandroupolis, Greece. [6]Lung and Regenerative Medicine Institutes, Cedars-Sinai Medical Center, Los Angeles, CA, USA. [7]Faculty of Health and Medical Sciences, University of Surrey, Guildford, UK. [8]Dept of Pathophysiology, Medical School, National and Kapodistrian University of Athens, Athens, Greece. [9]Institute of Pathology, Lausanne University Hospital, Lausanne, Switzerland. [10]4th Dept of Internal Medicine, Attikon University Hospital, University of Athens Medical School, Athens, Greece. [11]Hellenic Centre for Disease Control and Prevention, Athens, Greece. [12]Translational Epigenetics Group, Institute of Pathology, University Medical Center Göttingen, Göttingen, Germany. [13]Center for Molecular Medicine, University of Cologne, Cologne, Germany. [14]Oncogenomic and Epigenetic Unit, IRCCS, Regina Elena National Cancer Institute, Rome, Italy. [15]Faculty Institute for Cancer Sciences, Manchester Academic Health Sciences Centre, University of Manchester, Manchester, UK. [16]Center for New Biotechnologies and Precision Medicine, Medical School, National and Kapodistrian University of Athens, Athens, Greece. [17]Contributed equally. [18]P.J. Barnes and V.G. Gorgoulis contributed equally to this article as lead authors and supervised the work.

Corresponding author: Vassilis G. Gorgoulis (vgorg@med.uoa.gr)

Check for updates

Shareable abstract (@ERSpublications)
In severe COVID-19, alveolar type 2 (AT2) cells infected by SARS-CoV-2 exhibit senescence accompanied by a proinflammatory phenotype, a molecular mechanism that may be important in persistence of disease (post-acute sequelae of COVID-19) and mutagenesis https://bit.ly/3fnopg9

Cite this article as: Evangelou K, Veroutis D, Paschalaki K, *et al*. Pulmonary infection by SARS-CoV-2 induces senescence accompanied by an inflammatory phenotype in severe COVID-19: possible implications for viral mutagenesis. *Eur Respir J* 2022; 60: 2102951 [DOI: 10.1183/13993003.02951-2021].

This single-page version can be shared freely online.

Copyright ©The authors 2022.

This version is distributed under the terms of the Creative Commons Attribution Licence 4.0.

This article has an editorial commentary: https://doi.org/10.1183/13993003.01101-2022

Received: 27 Aug 2021
Accepted: 24 Dec 2021

Abstract

Background Severe acute respiratory syndrome coronavirus 2 (SARS-CoV-2) infection of the respiratory system can progress to a multisystemic disease with aberrant inflammatory response. Cellular senescence promotes chronic inflammation, named senescence-associated secretory phenotype (SASP). We investigated whether coronavirus disease 2019 (COVID-19) is associated with cellular senescence and SASP.

Methods Autopsy lung tissue samples from 11 COVID-19 patients and 43 age-matched non-COVID-19 controls with similar comorbidities were analysed by immunohistochemistry for SARS-CoV-2, markers of senescence and key SASP cytokines. Virally induced senescence was functionally recapitulated *in vitro*, by infecting epithelial Vero-E6 cells and a three-dimensional alveosphere system of alveolar type 2 (AT2) cells with SARS-CoV-2 strains isolated from COVID-19 patients.

Results SARS-CoV-2 was detected by immunocytochemistry and electron microscopy predominantly in AT2 cells. Infected AT2 cells expressed angiotensin-converting enzyme 2 and exhibited increased senescence ($p16^{INK4A}$ and SenTraGor positivity) and interleukin (IL)-1β and IL-6 expression. *In vitro*, infection of Vero-E6 cells with SARS-CoV-2 induced senescence (SenTraGor), DNA damage (γ-H2AX) and increased cytokine (IL-1β, IL-6, CXCL8) and apolipoprotein B mRNA-editing (APOBEC) enzyme

expression. Next-generation sequencing analysis of progenies obtained from infected/senescent Vero-E6 cells demonstrated APOBEC-mediated SARS-CoV-2 mutations. Dissemination of the SARS-CoV-2-infection and senescence was confirmed in extrapulmonary sites (kidney and liver) of a COVID-19 patient.

Conclusions We demonstrate that in severe COVID-19, AT2 cells infected by SARS-CoV-2 exhibit senescence and a proinflammatory phenotype. *In vitro*, SARS-CoV-2 infection induces senescence and inflammation. Importantly, infected senescent cells may act as a source of SARS-CoV-2 mutagenesis mediated by APOBEC enzymes. Therefore, SARS-CoV-2-induced senescence may be an important molecular mechanism of severe COVID-19, disease persistence and mutagenesis.
